# Selective Devaluation Affects the Processing of Preferred Rewards

**DOI:** 10.3758/s13415-021-00904-x

**Published:** 2021-04-30

**Authors:** Dana M. Huvermann, Christian Bellebaum, Jutta Peterburs

**Affiliations:** 1 Department of Biological Psychology, Institute of Experimental Psychology, Heinrich-Heine-University Düsseldorf, Universitätsstraße 1, Düsseldorf, 40225 Germany; 2 Department of Medicine, Medical Psychology, MSH Medical School Hamburg, Am Kaiserkai 1, Hamburg, 20457 Germany

**Keywords:** Reward value, Selective devaluation, P300, Feedback-related negativity, P2

## Abstract

The present study investigated whether the representation of subjective preferences in the event-related potential is manipulable through *selective devaluation*, i.e., the consumption of a specific food item until satiety. Thirty-four participants completed a gambling task in which they chose between virtual doors to find one of three snack items, representing a high, medium, or low preference outcome as defined by individual desire-to-eat ratings. In one of two test sessions, they underwent selective devaluation of the high preference outcome. In the other, they completed the task on an empty stomach. Consistent with previous findings, averaged across sessions, amplitudes were increased for more preferred rewards in the time windows of P2, late FRN, and P300. As hypothesised, we also found a selective devaluation effect for the high preference outcome in the P300 time window, reflected in a decrease in amplitude. The present results provide evidence for modulations of reward processing not only by individual factors, such as subjective preferences, but also by the current motivational state. Importantly, the present data suggest that selective devaluation effects in the P300 may be a promising tool to further characterise altered valuation of food rewards in the context of eating disorders and obesity.

**Supplementary Information:**

The online version contains supplementary material available at 10.3758/s13415-021-00904-x.

To cope in an ever-changing environment, we need to process adequately the consequences of our actions and associate one with the other. For instance, when deciding between different dishes in a restaurant, we need to take into account which dish matches our appetite best and whether it was consistently prepared to our liking in the past. We generally evaluate the utility of our choices and actions based on outcome attributes such as probability, valence, or magnitude. Because outcome processing comprises a fast-paced sequence of processing steps (Glazer et al., 2018), the underlying neural mechanisms have been extensively researched using measures with a high temporal resolution, and in research with humans electroencephalography (EEG) is the most frequently applied method.

An early component of the event-related potential (ERP) that has been associated with outcome processing is the feedback-related negativity (FRN) (Miltner et al., 1997). The FRN, a frontocentral negativity peaking between 200 and 300 ms after outcome presentation (Glazer et al., 2018), is typically more pronounced for undesirable compared with desirable outcomes and has been linked to performance and feedback evaluation (Bellebaum, Kobza, et al., 2010a; Holroyd & Coles, 2002; Miltner et al., 1997; Nieuwenhuis et al., 2004; Wu & Zhou, 2009). It is generated in the anterior cingulate cortex (ACC; Gehring & Willoughby, 2002; Miltner et al., 1997; Ruchsow et al., 2002; Zhou et al., 2010), a structure that receives mesocortical projections from dopamine neurons in the substantia nigra (Emson & Koob, 1978; Lindvall et al., 1974; Porrino & Goldman-Rakic, 1982) and ventral tegmental area (Beckstead et al., 1979; Porrino & Goldman-Rakic, 1982; Swanson, 1982). Based on these findings, Holroyd and Coles (2002) assumed that the FRN is driven by dopaminergic activity. Interestingly, midbrain dopaminergic neurons code a reward prediction error: while an unexpected reward is answered with increased dopaminergic activity, an expected reward is answered with no such increase, and an expected but omitted reward results in a decrease in activity compared to baseline (Schultz et al., 1997; Zaghloul et al., 2009). Moreover, midbrain dopaminergic activity is also modulated by reward magnitude, with a reward smaller or larger than expected eliciting suppression or activation, respectively, relative to baseline (Tobler et al., 2005).

Accordingly, the FRN has been shown to be affected by both outcome probability and magnitude. It is more negative for unexpected compared to expected negative outcomes, and more positive for unexpected compared to expected positive outcomes (Hajcak et al., 2007; Potts et al., 2006; Walsh & Anderson, 2011). Similarly, the negativity is more pronounced for smaller compared to larger amounts of monetary rewards (Holroyd et al., 2004; Kreussel et al., 2012; Wu & Zhou, 2009), and the amplitude difference between positive and negative outcomes is larger with higher (potential) outcomes (Bellebaum, Polezzi, & Daum, 2010b). Along these lines, the FRN is thought to reflect dopaminergic activity, thus also coding a reward prediction error (Pfabigan et al., 2011; Sambrook & Goslin, 2015; Wu & Zhou, 2009). This notion has been confirmed by means of single-trial analyses (Burnside et al., 2019; Fischer & Ullsperger, 2013). In recent years, there has been a debate whether effects in the FRN difference signal between positive and negative feedback are primarily driven by a negativity in response to negative outcomes or by a positivity in response to rewards. It has been proposed that positive prediction errors elicit a relative positivity in the signal, the reward positivity (Becker et al., 2014; Holroyd et al., 2008; Proudfit, 2015). This has been supported by studies using principal component analysis (Foti et al., 2011) as well as by combined EEG and fMRI studies (Becker et al., 2014; Carlson et al., 2011).

A later ERP component that has been implicated in outcome processing is the P300 (Bellebaum, Polezzi, & Daum, 2010b; Yeung & Sanfey, 2004), a positive deflection peaking between 300 and 600 ms after outcome presentation (Glazer et al., 2018) that typically increases in amplitude from frontal to parietal sites (Johnson, 1993). While its neural origin has not been fully determined (Glazer et al., 2018; Huang et al., 2015; Polich, 2007), it has been associated with dopaminergic midbrain structures (Andreou et al., 2013; Pfabigan et al., 2014; Rule et al., 2002). Although findings concerning the P300’s sensitivity to outcome valence have been inconsistent (Bellebaum, Polezzi et al., 2010; Foti & Hajcak, 2012; Gu et al., 2011; Hajcak et al., 2007; Kobza et al., 2011; Kreussel et al., 2012; Polezzi et al., 2010), coding of outcome magnitude in the P300 has been reliably shown, with more positive amplitudes for larger outcomes (Bellebaum, Polezzi et al., 2010; Goyer et al., 2008; Gu et al., 2011; Kreussel et al., 2012; Meadows et al., 2016; Polezzi et al., 2010; Sato et al., 2005; Wu & Zhou, 2009; Yeung & Sanfey, 2004). The P300 also shows relatively consistent effects of outcome probability, with more positive P300 amplitudes for unexpected compared to expected outcomes (Bellebaum & Daum, 2008; Hajcak et al., 2005; Hajcak et al., 2007; Wu & Zhou, 2009; but also see Kobza et al., 2011; Kreussel et al., 2012).

Functionally, the P300 has been subdivided into an earlier, attention-focussed component in frontal areas (P3a) and a later, memory-focussed component in centroparietal areas (P3b; Polich, 2007). It has been suggested that the P300 may reflect neural activity that inhibits other, unrelated processes and enhances attentional focus to promote memory storage for the currently focussed stimulus (Polich, 2012). Along these lines, the P300 has been shown to increase in amplitude with higher motivational significance of stimuli (Begleiter et al., 1983; Nieuwenhuis et al., 2005; San Martín, 2012; Severo et al., 2020).

Consistent with reward magnitude effects in both FRN and P300, Peterburs et al. (2019) recently reported that subjective reward preferences, corresponding to subjective reward magnitude, had a significant impact on the outcome-locked ERP in the time windows of FRN and P300, with more positive amplitudes for a preferred relative to a less liked food reward. Preference coding started as early as 170 ms after outcome onset and thus in the time window of the P2, an early positive ERP component that also has been shown to code outcome magnitude (Flores et al., 2015; Potts et al., 2006; San Martín et al., 2010). However, the neural representation of subjective preferences and their interplay with motivational states (Balleine & Dickinson, 1998) is still largely unclear. A previous study by Baker et al. (2016) reported that after a period of abstinence, smokers showed a more positive FRN (operationalised as the reward-no reward difference) for cigarette compared to monetary rewards. This finding suggests that the induction of craving by the period of abstinence altered the motivational value of outcomes and their respective processing endeavour. While Baker et al.’s study offers a first glimpse into dependence of subjective reward processing on the current motivational state, the study lacked in control conditions; it did not include a nonabstinence condition or a nonsmoker control group. In the present study, we aimed to investigate whether motivational state-induced shifts of subjective reward preferences are reflected in neural processing of the rewards in a fully controlled design that does not entail substance addiction.

For that purpose, we applied selective devaluation, also referred to as selective satiety (Rolls et al., 1981), a procedure in which one item of a variety of food items is devalued through consumption. Changes in the (neural) processing of this food item can be attributed solely to a change of subjective valuation, as all physical attributes of the food item remain the same. fMRI studies on subjective devaluation have yielded consistent results: consumption of a food item reduced activation in the orbitofrontal cortex (OFC) associated with the consumed item, but not others (Gottfried et al., 2003; Howard & Kahnt, 2017; Kringelbach et al., 2003; O'Doherty et al., 2000; Valentin et al., 2007). It is, however, unclear whether selective devaluation interacts with subjective preferences for specific rewards during outcome processing. Importantly, when referring to subjective preferences, we mean long-term preferences that shift only slightly over the time span of few years (Nicklaus et al., 2004; Skinner et al., 2002) as described by Rozin (1990).

In the present study, we aimed to clarify whether the selective satiety effect extends to the manipulation of subjective preferences and how it is represented in the ERP. We tested a sample of healthy adults both with and without applying selective devaluation of a preferred outcome, using a variant of the gambling task introduced by Peterburs et al. (2019), but with individually determined rewards of high, medium, and low preference. We hypothesised that selective devaluation of the high preference reward would affect the amplitude within the time windows of the P2, FRN, and P300 ERP components, such that preference coding in the ERP is maintained without selective devaluation but altered when selective devaluation is applied. More specifically, after selective devaluation, the ERP amplitude should be reduced in response to the high preference outcome, so that processing differences compared with the medium and low preference outcome are reduced.

## Methods

### Participants

Thirty-four healthy adults (24 women) with a mean age of 22.2 years (*SD* = 3.7 years; age from 18 to 34 years) participated in the present study. All participants reported no history of neurological or psychiatric disorders, no intake of medication affecting the central nervous system and normal or corrected-to-normal vision. Four participants stated to be left-handed, one to be ambidextrous, and the remaining 29 stated to be right-handed. Two participants had to be excluded due to noisy EEG data and one participant had to be excluded due to an extreme preference decline for the high preference outcome from the first to the second appointment. While this participant chose the highest possible rating for the high preference outcome in the first session (9), they chose the lowest possible rating for the high preference outcome in the second appointment (1) and expressed that this was to make sure that they would not be offered the same snack item again in the second appointment. Because such behaviour was not present in or expressed by any other participant, this subject’s data were excluded from the analyses.

The required sample size was estimated via G*Power (Faul et al., 2007; Faul et al., 2009). Because G*Power does not offer an approach to estimate sample sizes for cluster-based permutation analyses, we estimated sample size by using the effect size from the interaction of condition (active vs. observational) and outcome type (high, medium, or low preference) measured in the P300 within the repeated measure ANOVA in Peterburs et al. (2019). This setup was most similar to our study (with the factor agency—active vs. observational—instead of valuation condition), and they conducted both an ANOVA as well as a cluster-based permutation analysis. Assuming a similar effect size of η_p_^2^ = 0.16 and keeping conventional levels of α = 0.05 and Power = 0.80, a sample size of 30 participants was required. All participants gave informed, written consent before their participation. This study was approved by the ethics committee of the Faculty of Mathematics and Natural Sciences at Heinrich-Heine-University Düsseldorf and conforms to the Declaration of Helsinki.

### Stimuli

Throughout the study, participants had to give desire-to-eat ratings for nine snack items and hunger ratings (see the *Procedure* section for details on when the ratings were taken). Ratings were given on a scale from 1 to 9, with 1 indicating absolutely no current desire to eat the snack/no sensation of hunger, and 9 indicating a very high current desire to eat the snack/a very high sensation of hunger. The snack items used in the present study belonged to three categories: sweet (gummi bears, milk chocolate, candy-coated chocolate drops); savoury (crisps, salted peanuts, salted mini pretzels); and neutral snacks (edible wafer paper, rice cake, crispbread). Of the nine rated snack items, three were chosen to be used in the experimental task individually for each participant. The item with the highest desire-to-eat rating among the sweet and savoury snacks in the pre-rating (i.e., at the beginning of the first experimental session) was chosen as the high preference outcome (HPO). A snack of the other category (sweet or savoury, respectively) that was rated lower than the HPO but still in a medium range of the overall ratings of the participant was chosen as the medium preference outcome (MPO). Only in one case did we have to choose one of the neutral items as an MPO, as preference formation would not have been possible otherwise. The low preference outcome (LPO) was chosen as the item with the highest ratings among the neutral items that still had a lower rating than the HPO and MPO.

### Procedure

Before data acquisition, participants had to fill in an online questionnaire to assess whether they would qualify for the study in terms of health and palatability of the snacks. To this end, participants needed to state whether they suffered from an eating disorder (or any neurological or psychiatric disorder), diabetes, a disorder or absence of their sense of taste or smell, whether they recently started a diet or intended to begin one before the appointments, whether they had any allergies/intolerances against any of the snacks, and to which degree they liked the snack items (confer Friedel et al., 2014; Horstmann et al., 2015; Janssen et al., 2017; Tricomi et al., 2009). To ensure preference formation was possible, we only admitted participants who could safely eat and liked at least one of the sweet and one of the savoury items (as indicated by a rating above 5 on a scale from 1 to 9 where 1 indicated a strong disliking and 9 indicated a strong liking of the item). Note that the actual food items serving as HPO, MPO, and LPO were chosen based on the desire-to-eat ratings that were conducted in the lab (see above).

To avoid systematic effects of fatigue and minimise repetition effects for the experimental task, we decided to deviate from the typical pre-/post-devaluation design (confer Horstmann et al., 2015; Soares et al., 2012; Valentin et al., 2007) and recorded EEG after devaluation and nondevaluation in two separate appointments which took place at least 5 days apart, with a mean of 11.3 days between appointments (*SD* = 6.3 days; range 5-25 days). One of the appointments included a devaluation of the HPO before the experimental task, thus forming the devaluation condition, whereas the other one did not include such a devaluation and thus formed the nondevaluation condition. The order of the appointment types was counter-balanced across participants. In accordance with the procedure applied in previous studies (confer Friedel et al., 2014; Howard & Kahnt, 2017; Tricomi et al., 2009; Valentin et al., 2007), participants were instructed to fast for at least 6 hours before each appointment to encourage the consumption of the snack items through increased hunger and attractiveness of the snack items (Uher et al., 2006). On average, subjects declared to have fasted for 11.97 hours (*SD* = 4.16 hours; range 5.42-20 hours).

After participants arrived at the lab, they filled out a demographic questionnaire and a questionnaire inquiring about their current desire for the nine depicted snacks as well as their current hunger level (confer Horstmann et al., 2015; Howard & Kahnt, 2017; Soares et al., 2012; Valentin et al., 2007). We additionally took their statement of their height and weight for an exploratory analysis using the body mass index (BMI), which can be found in the supplementary material (see Supplementary Analysis 1). Subsequently, the preparation for the EEG took place while participants could eat the snack provided to them on the devaluation appointment and, to increase snacking behaviour (Lyons et al., 2013), watch a TV show or film of their choice via a provided streaming service.

To devaluate the HPO, a bowl containing 100 g of the respective HPO snack item was placed in front of the participants and they were asked to eat until they felt satiated or until they no longer wanted to eat the specific snack (confer Janssen et al., 2017 ; Soares et al., 2012 ; Tricomi et al., 2009 ; Valentin et al., 2007). They were encouraged to ask for another serving if the above-mentioned conditions were not met after finishing the previous serving. In that case, the bowl was refilled with another 100 g of the respective snack. Devaluation was quantified by weighing the snack bowl before and after each serving (Friedel et al., 2014; Horstmann et al., 2015) and through questionnaires inquiring about participants’ current desire to eat each of the nine snacks and current feeling of hunger before and after devaluation took place (or in the nondevaluation condition, before and after EEG preparation took place). Devaluation led to a significant reduction in hunger ratings (*M*_*diff*_ = −3.0, *SD*_*diff*_ = 1.9) compared with the nondevaluation condition (*M*_*diff*_ = 0.4, *SD*_*diff*_ = 0.8), *t*(30)= 9.08, *p* < 0.001. On average, participants consumed 99.1 g (*SD* = 56.6 g) or 454.9 kcal (*SD* = 245.0 kcal). Participants could determine the time for eating the snack by themselves and were considered satiated once they quit food consumption (Janssen et al., 2017; Soares et al., 2012; Tricomi et al., 2009; Valentin et al., 2007). Experimenters sat behind a room separator whenever EEG preparation was finished but snacking continued. Subsequently, participants were educated about EEG artefacts and how to reduce them, followed by the standardised instructions for the experimental task.

**Fig. 1:**
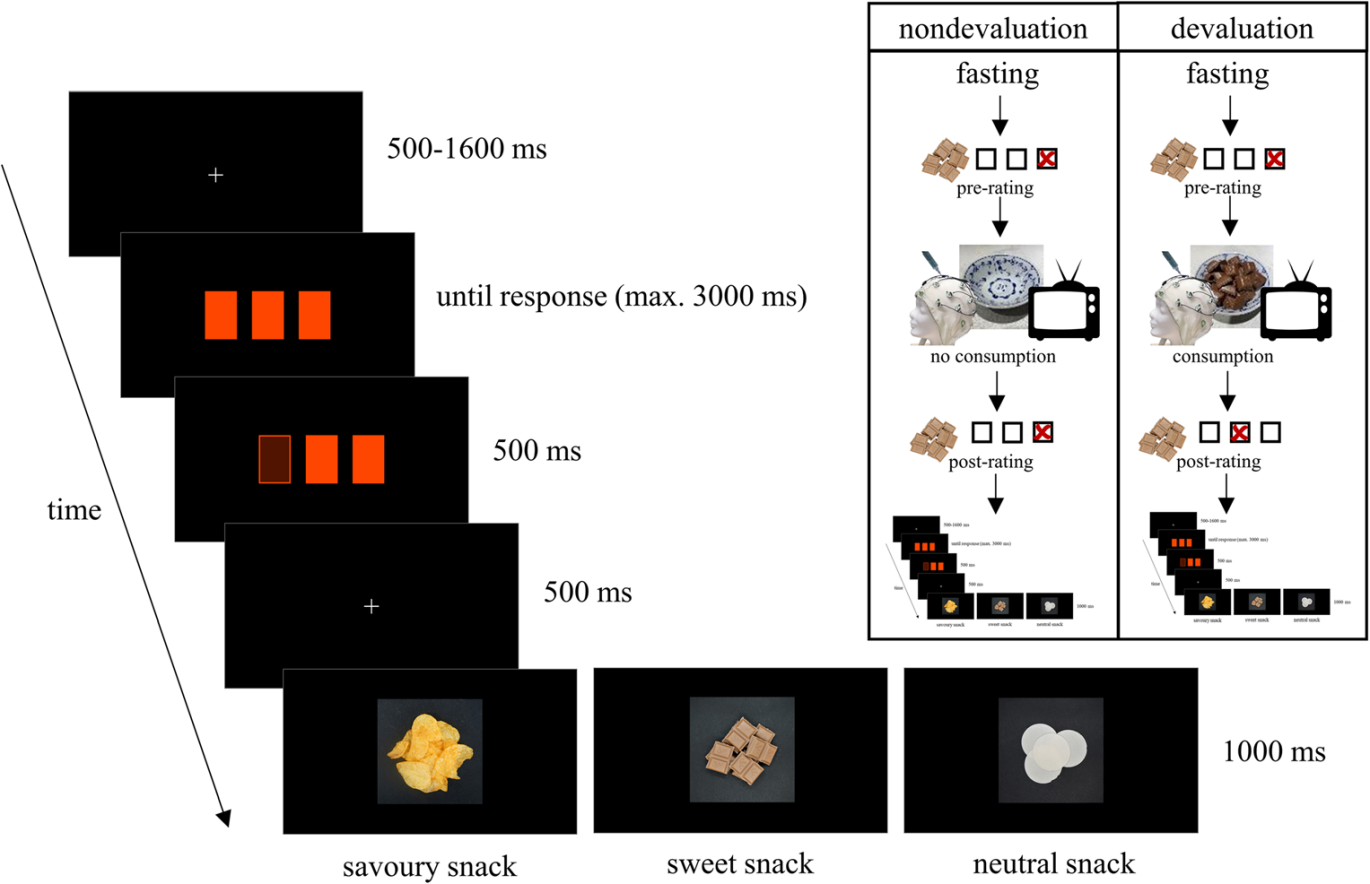
Sequence and time course of stimulus presentation in one trial of the gambling task as well as outline of the general experimental procedure

The experimental task consisted of three blocks of 100 trials, resulting in a total of 300 trials per session. Between blocks, participants had the opportunity to take breaks whose length they could determine by themselves. During each trial, participants initially saw three rectangles representing doors of which they could choose one by pressing one of three buttons on a response box (Cedrus RB-740; Cedrus Corporation, San Pedro, CA) to find one of the three snacks “hidden” behind it. Figure 1 shows the procedure for one trial. After the presentation of a fixation cross for a randomly determined duration between 500 and 1600 ms, doors were shown until button press or for a maximum of 3,000 ms. After button press, the respective door was highlighted for 500 ms, followed by a fixation cross for 500 ms and then the display of one of the three snacks for 1,000 ms. Participants were told that they would win the snack they would discover most often. However, unbeknown to the subjects each snack was shown equally often and in randomised order. Stimuli were presented on a BenQ Senseye LED 27” monitor via Presentation (Version 20.0, Neurobehavioral Systems Inc., Albany, CA) on a SilverStone PC running Windows 10. Experimenters sat behind a room separator for the duration of the experimental task, which lasted approximately 25 minutes. After task completion, participants were handed their compensation at the second appointment and dismissed. Compensation consisted of course credit and the participants’ preferred snack.

### EEG recording and preprocessing

EEG was recorded from 28 active Ag/AgCl electrodes (F7, F3, Fz, F4, F8, FC5, FC1, FC2, FC6, T7, C3, Cz, C4, T8, CP5, CP1, CP2, CP6, P7, P3, Pz, P4, P8, PO9, O1, Oz, O2, PO10) that were positioned on an actiCap (BrainProducts GmbH, Munich, Germany) and arranged according to the 10-20 system (Chatrian et al., 1985). Two electrodes were attached to the mastoids. FCz served as online reference, and the ground electrode was installed at site AFz. To account for eye movement artefacts, two additional electrodes were attached: one horizontal electrooculogram (hEOG) electrode to the outer canthus of the left eye and one vertical electrooculogram (vEOG) electrode above the left eye. Impedances were kept below 5 kΩ. Data were sampled at a rate of 1,000 Hz, amplified with a BrainAmp DC amplifier (BrainProducts GmbH, Munich, Germany), and recorded with BrainVision Recorder software (Version 1.20.0506, BrainProducts GmbH, Munich, Germany) on an Intel Premium PC running Windows 10.

During preprocessing, the signal was first re-referenced to the mean signal of the mastoid electrodes, and the signal at FCz was restored. Next, we applied direct current (DC) detrending and a Butterworth filter with a low cutoff of 0.1 Hz (time constant: 1.59), a high cutoff of 30 Hz, and a notch filter of 50 Hz. We subsequently corrected for oculomotor artefacts using ocular correction independent component analysis (ICA) of the EEG data as implemented in BrainVision Analyzer 2 software, based on hEOG and vEOG for each participant, eliminating three to eight components via ICA backward transformation. Data was then segmented into epochs of 800 ms, starting 200 ms before outcome onset and ending 600 ms after outcome onset for each of the three outcomes. Next, a baseline correction was applied based on the 200 ms immediately preceding outcome onset. Segments with a maximum difference of values over 100 μV or an activity lower than 0.1 μV within an interval of 100 ms, a voltage step exceeding 50 μV/ms, or values above 100 μV or below −100 μV were then rejected using automatic artefact rejection. On average, 96.7 segments per outcome type and valuation condition remained (SD = 4.8 segments). Last, data were averaged according to valuation condition and outcome type.

### Data Analysis

#### Behavioural data

To confirm the classification into outcome types and the behavioural selective devaluation, a repeated measure analysis of variance (ANOVA) for the desire-to-eat ratings with the within-subject factors valuation condition (devaluation, nondevaluation), outcome type (LPO, MPO, HPO), and time point (pre, post) was conducted. An alpha level of *p* < 0.05 was considered statistically significant. In case of violation of sphericity, degrees of freedom were adjusted following the Greenhouse-Geisser method. Pairwise comparisons following significant effects of outcome type were Bonferroni-corrected with an α-level of *p* = 0.017. Significant interactions were resolved by subordinate ANOVAs and post-hoc testing as specified below.

#### Cluster-based permutation analysis of ERP data

As in this study, we hypothesized to find effects in a larger time window spanning multiple ERP components (P2, FRN, and P300) with differing spatial distributions, we employed a cluster-based permutation analysis as implemented in the FieldTrip toolbox (version 20200409, www.fieldtriptoolbox.org; Oostenveld et al., 2011) to determine the spatiotemporal differences in the processing of the outcome types in the different valuation conditions. Note that this procedure also was used in a prior study on reward preferences by our group using the same task (Peterburs et al., 2019). Cluster-based permutation analysis is a nonparametric method to test for differences between experimental conditions in data with a high-dimensional spatiotemporal structure, such as EEG, while correcting for multiple comparisons prevalent in such data (Maris & Oostenveld, 2007). Because it does not rely on a priori selection of electrodes and time windows, it offers a more objective approach than classical ERP analyses which rely on challengeable choices of electrodes and time windows based on visual inspection and/or previous literature. We analysed data in several steps to account for our 2 x 3 experimental design, as the method does not allow us to consider more than one experimental factor at a time (Peterburs et al., 2019).

Data were down-sampled to 100 Hz. In a first step, we wanted to assess general effects of subjective preferences on outcome processing. To this end, we pooled the two valuation conditions by averaging the ERPs per participant for each outcome type (HPO, MPO, and LPO) across the devaluation and nondevaluation conditions. Univariate ANOVAs were performed for each data point and electrode in the down-sampled data, starting at outcome onset and ending 600 ms after outcome onset. Samples were considered significant if less than 0.05 of reference test statistics exceeded the test statistic observed in the sample. Reference test statistics were calculated from 1000 samples generated by means of random permutation, i.e., randomly shuffling data. If at least three spatially and temporally adjacent samples fulfilled this criterion, they were combined into a cluster. Concerning spatial adjacency, a neighbouring distance of 0.225 was used (yielding four to 17 neighbours per electrode, *M* = 10.88, *SD* = 3.74), and the acticap-64ch-standard2.mat 2D template (FieldTrip, version 20200409) was applied. The sum of the *F*-values of all samples in a cluster was used as a test distribution for the second-layer cluster statistic. The *p*-value for each cluster was estimated by comparing the test distribution with a reference distribution that was again calculated by random permutations as described above.

To clarify which of the three outcomes differed from each other, we conducted analogous cluster-based permutation analyses based on dependent-sample *t*-tests for the outcome types (HPO vs. LPO; MPO vs. LPO; HPO vs. MPO). While these analyses were conducted for the same time period as the previously described analysis for the overall main effect (from outcome onset until 600 ms after), only effects within the spatiotemporal clusters of the main effect were considered and interpreted.

To explore the interaction effect of outcome type and valuation condition, which was of particular interest as it addressed our hypothesis, the same procedure as for the cluster-based permutation analysis of the main effect was applied to the devaluation–nondevaluation difference signals that were created for each outcome type. Hence, we again obtained three spatiotemporal patterns of activity. Here, significant clusters indicated that the difference between the devaluation and nondevaluation condition differed as a function of outcome type. To resolve the interaction, separate follow-up cluster-based analyses using *F*-tests were performed to find processing differences between the three outcome types for each valuation condition separately. In case of significance, we conducted post-hoc, cluster-based permutation analyses based on *t*-tests for each outcome type and valuation condition, to further discern which outcome types differed significantly from one another. Again, clusters found in these post-hoc tests are only reported and considered if they coincided with the time windows of the difference signal main effect clusters. To account for the bi-directionality of this test, we chose an alpha-level of 0.025 to determine significance.

## Results

### Behavioural Results

**Fig. 2:**
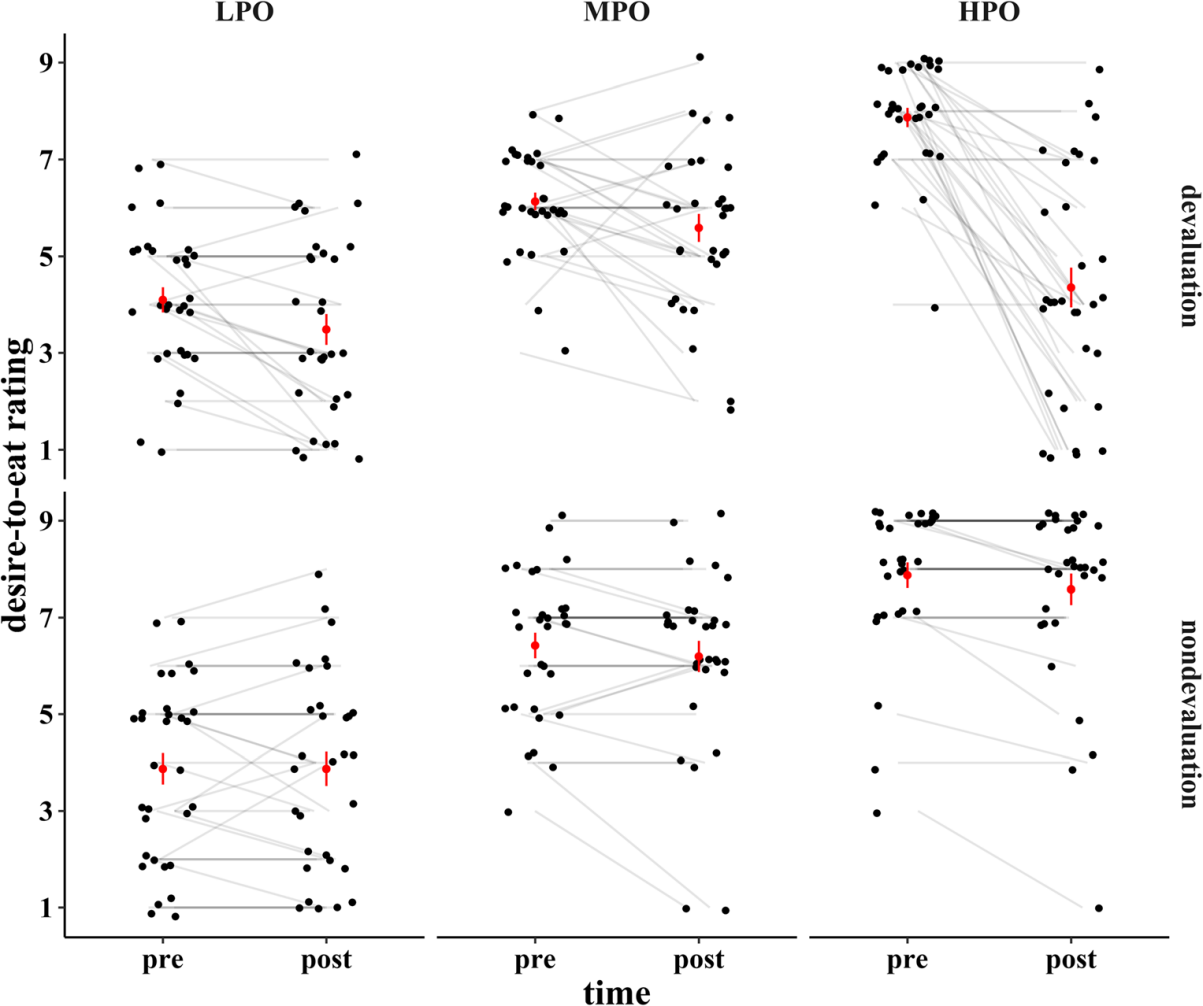
Desire-to-eat ratings per outcome type and valuation condition for two different time points. Note that pre and post refers to the time point of devaluation in the devaluation condition and to the time point of EEG preparation in the nondevaluation condition. Mean and standard error are displayed in red. HPO = high preference outcome; MPO = middle preference outcome; LPO = low preference outcome

For the desire-to-eat ratings (Fig. 2), a triple interaction between outcome type, valuation condition, and time point could be observed, *F*(2, 60) = 24.24, *p* < 0.001, η_p_^2^ = 0.45. Because our main interest lay in the selective devaluation effect, we investigated only this triple interaction further. The complete analysis is reported in Table 1. The triple interaction was resolved by separate within-subject ANOVAs for both time points.

**Table 1:** Complete inferential statistics for the within-subject time point x valuation condition x outcome type ANOVA

Effect	*F*	*df*	*p*	η_p_^2^
Time point	48.72	1, 30	<0.001	0.62
Valuation condition	8.99	1, 30	0.005	0.23
Outcome type	61.02	2, 60	<0.001	0.67
Time point * valuation condition	37.43	1, 30	<0.001	0.56
Time point * outcome type	39.09	1.62, 48.65	<0.001	0.57
Valuation condition * outcome type	12.46	2, 60	<0.001	0.29
Time point * valuation condition * outcome type	24.24	2, 60	<0.001	0.45

*Note*. *n* = 31.

For the pre-ratings, no effect of valuation condition, *F*(1, 30) = 0.13, *p* = 0.909, and neither an interaction of valuation condition and outcome type could be found, *F*(2, 60) = 1.15, *p* = 0.322. Instead, a main effect of outcome type emerged, *F*(2, 60) = 102.93, *p* < 0.001, η_p_^2^ = 0.77. Post-hoc *t*-tests yielded that all outcome types differed significantly from one another, all *p* < 0.001, with the highest rating for the HPO, followed by the MPO and then LPO, reflecting the intended preference structure.

For the post-ratings, the interaction between valuation condition and outcome type reached significance, *F*(2, 60) = 25.58, *p* < 0.001, η_p_^2^ = 0.46. This interaction was then resolved by conducting follow-up ANOVAs separately for each valuation condition. The outcome type effect within the nondevaluation condition reached significance, *F*(2, 60) = 47.99, *p* < 0.001, η_p_^2^ = 0.62. Post-hoc *t*-tests yielded that all outcome types differed significantly from one another, all *p* ≤ 0.006, with the highest rating for the HPO, followed by the MPO and then LPO, reflecting the intended preference structure. The outcome type effect within the devaluation condition also reached significance, *F*(2, 60) = 12.44, *p* < 0.001, η_p_^2^ = 0.29. Post-hoc *t*-tests showed, however, that the underlying pattern was different. The MPO now reached the highest desire-to-eat ratings and was rated significantly higher than the LPO, *p* < 0.001, and the HPO, *p* = 0.029, while ratings for the LPO and HPO did not differ significantly, *p* = 0.217.

For an additional resolution of the triple interaction, we conducted ANOVAs with the factors time point and outcome type separately for the valuation conditions. For the nondevaluation condition, the interaction of time point and outcome type did not reach significance, *F*(2, 60) = 1.28, *p* = 0.287. However, the main effect of outcome type reached significance, *F*(2,60) = 63.21, *p* < 0.001, η_p_^2^ = 0.68, as well as the main effect of time point, *F*(1, 30) = 4.61, *p* = 0.040, η_p_^2^ = 0.13. All outcome types differed significantly from one another, all *p* ≤ 0.002; the highest rating was for the HPO, followed by the MPO, and then the LPO. Concerning time points, ratings were slightly lower for the post-rating (*M* = 5.9, *SD* = 1.2) compared with the pre-rating (M = 6.1, SD = 1.5). For the devaluation condition, an interaction between time point and outcome type emerged, *F*(1.61, 48.22) = 37.86, *p* < 0.001, η_p_^2^ = 0.56. Follow-up repeated-measures ANOVAs with outcome type as the single factor, separately for the time points, yielded a significant outcome type effect for both the pre-ratings, *F*(2, 60) = 75.90, *p* < 0.001, η_p_^2^ = 0.72, and the post-ratings, *F*(2, 60) = 12.44, *p* < 0.001, η_p_^2^ = 0.29. While for the pre-ratings, all outcome types differed significantly from one another, all *p* < 0.001, following the same pattern as the nondevaluation condition (HPO > MPO > LPO), HPO and LPO no longer differed significantly for the post-ratings, and the MPO was rated higher than both HPO and LPO (see above for *p*-values). This result pattern thus directly reflects the selective devaluation effect.

### Cluster-based Permutation Analysis

**Fig. 3:**
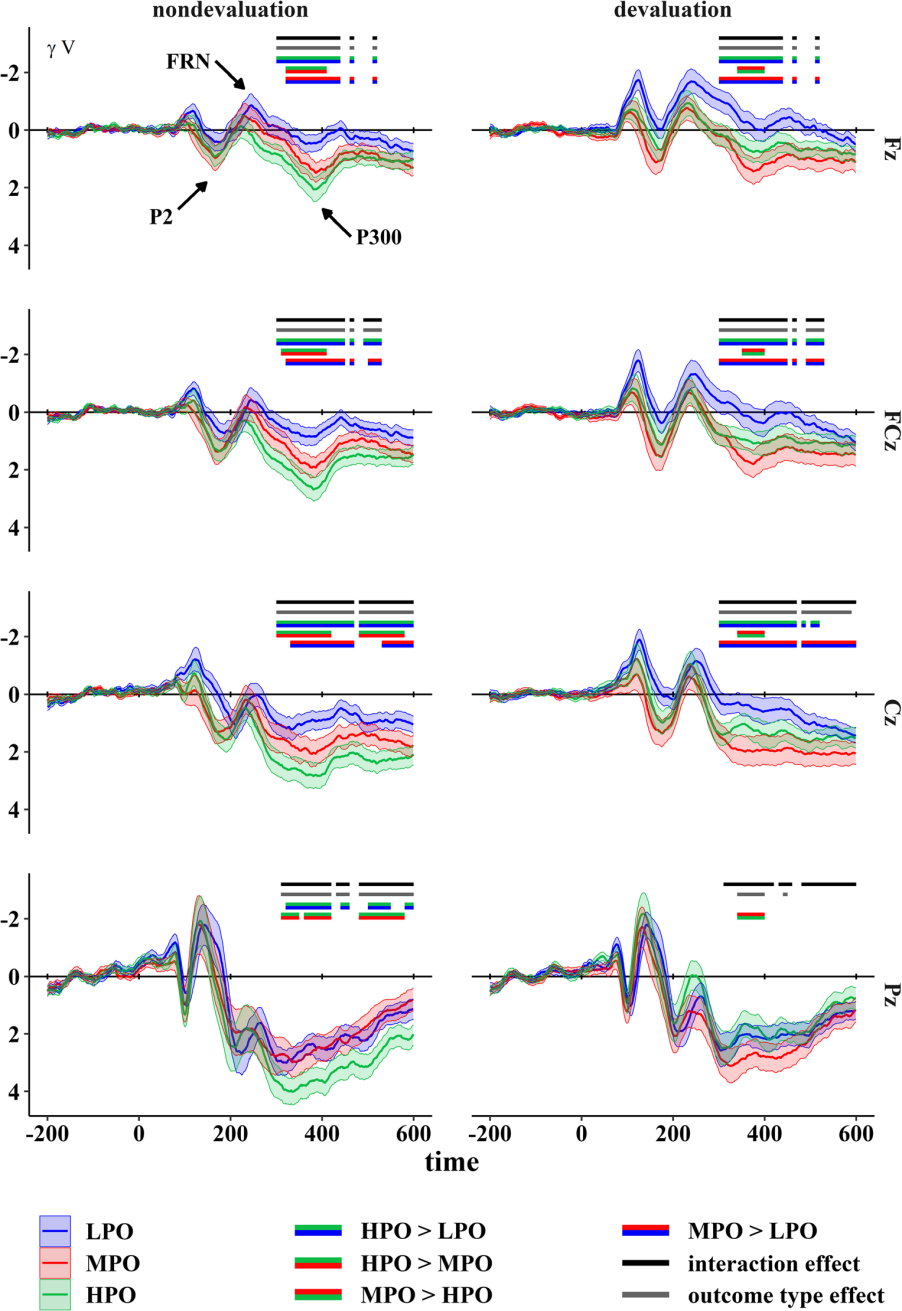
ERPs elicited by outcome presentation as a function of outcome type (HPO = high preference outcome, MPO = medium preference outcome, LPO = low preference outcome) and valuation condition (devaluation, nondevaluation). Lines above grand averages mark time windows with significant effects. Standard errors are displayed as ribbons around the lines

Outcome-locked grand averages according to outcome type and valuation condition at midline electrodes (Fz, FCz, Cz, and Pz) are displayed in Fig. 3.

#### Main Effect of Outcome Type

**Fig. 4:**
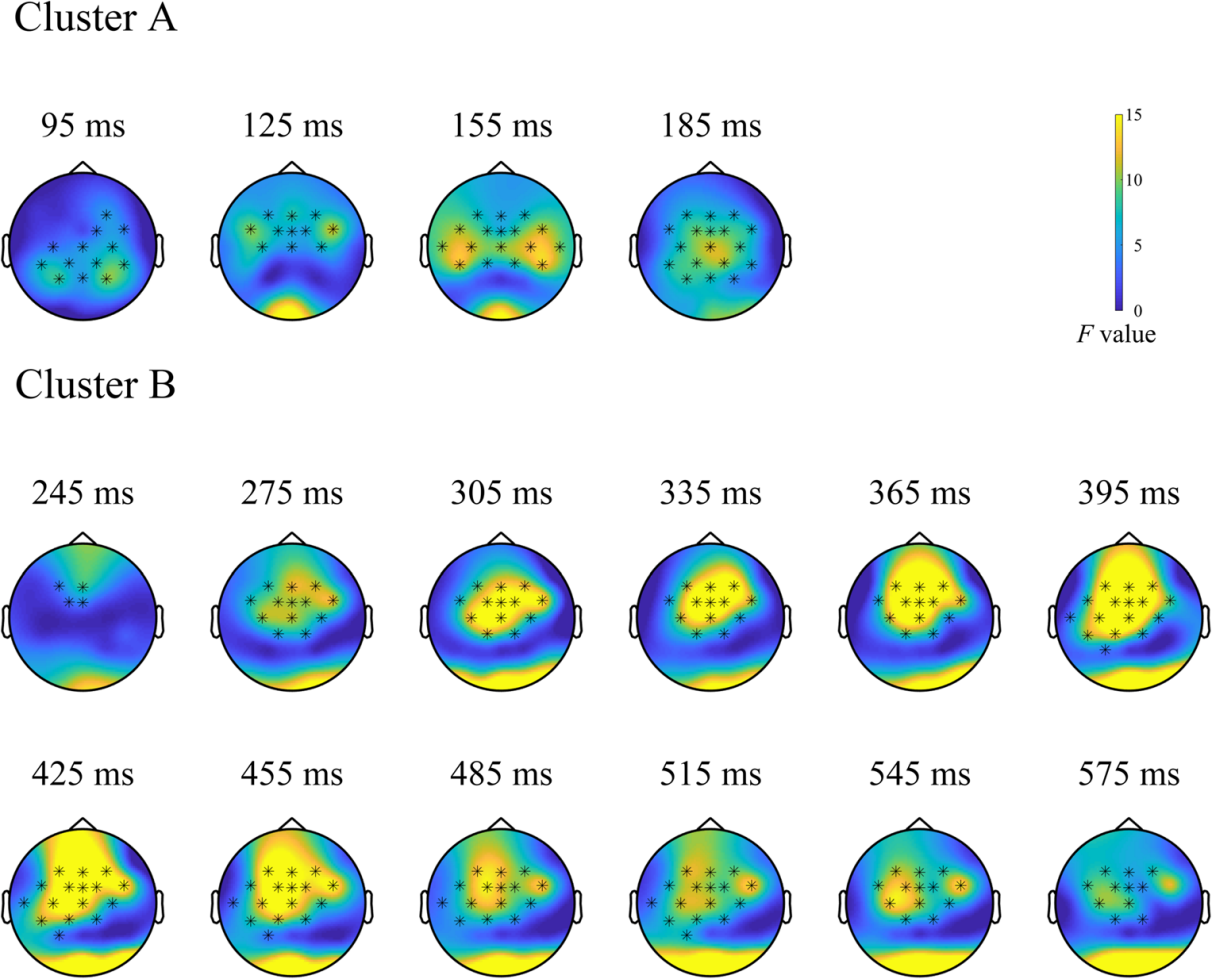
Clusters (90-200 and 240-600 ms) showing significant modulation of outcome processing by outcome type. Topographical plots represent time series of *F*-values at each channel at the respective time point. Stars indicate electrodes included in the cluster

Cluster-based permutation tests for the outcome type main effect revealed significant differences between outcome types (*p* < 0.001). This effect was evident within two separate clusters (for topographical plots, see Fig. 4 or Supplementary Figure S2 for a more detailed version): The earlier cluster covered a time window from 90 to 200 ms and included frontal, frontocentral, central, centroparietal, parietal, and occipital electrodes. Frontal, frontocentral, and central electrodes were most consistently involved. This cluster thus roughly corresponds to spatiotemporal attributes of the N1 and P2. The later cluster covered a time window ranging from 240 to 600 ms and spanned over frontal, frontocentral, central and (centro)parietal electrodes. Frontal, frontocentral, and central electrodes were most consistently included, suggesting that this cluster captures the late FRN and the P300 time window.

Subsequently, follow-up, cluster-based analyses with dependent-sample *t*-tests were conducted for each comparison of outcome types. Comparisons between HPO and LPO as well as between MPO and LPO matched findings of the main effect closely: the amplitude was significantly more positive for the HPO compared with the LPO (*p* < 0.001). This effect manifested in two clusters covering time windows from 90 to 200 ms and from 240 to 600 ms spanning over frontal, frontocentral, central, centroparietal, and parietal electrodes (for topographical plots, see Supplementary Figure S3). For the comparison of MPO and LPO, the MPO yielded a more positive amplitude compared to the LPO (*p* < 0.001) as well. This effect corresponded to an earlier cluster that covered the time window between 90 and 200 ms and included frontal, frontocentral, central, centroparietal, and parietal electrodes. A later cluster from 240 to 600 ms included frontal, frontocentral, central, and centroparietal electrodes (for topographical plots, see Supplementary Figure S4). The comparison between HPO and MPO did, however, not reach significance, *p* = 0.232.

#### Outcome type by valuation condition interaction

**Fig. 5:**
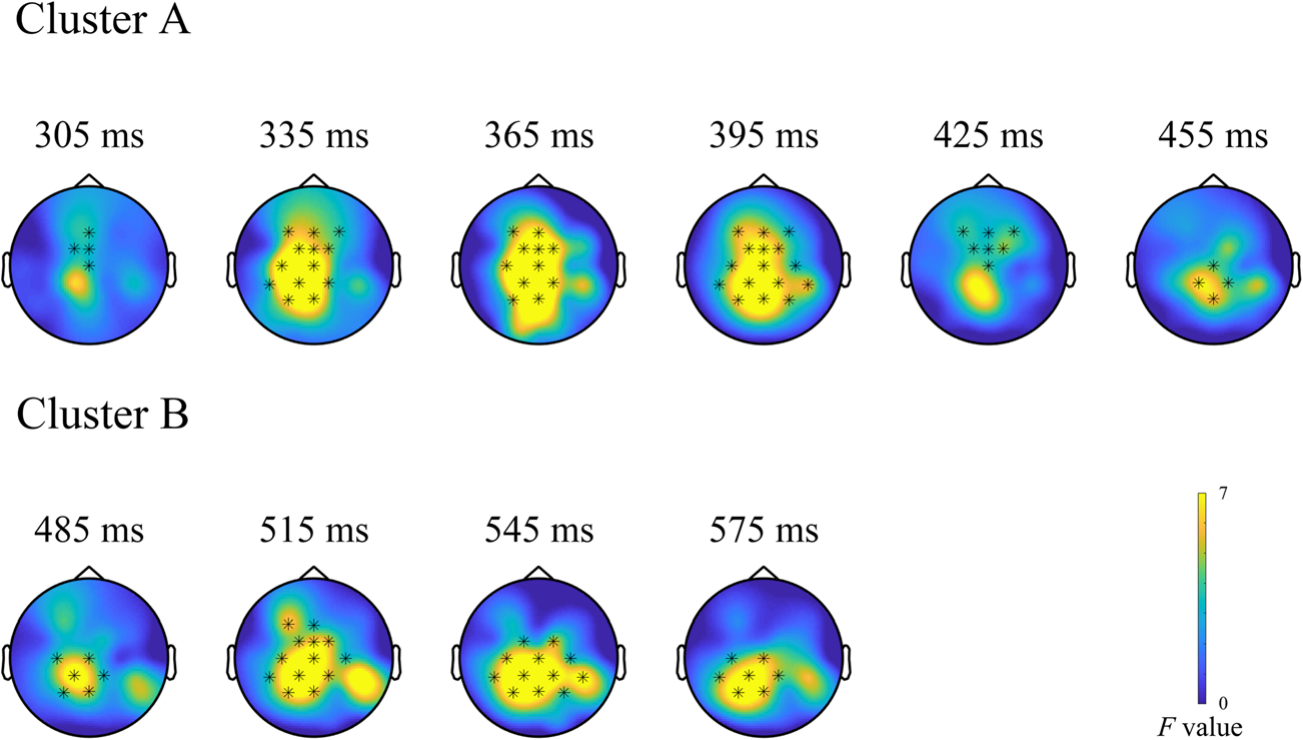
Clusters (300-470 and 480-600 ms) showing significant modulation of outcome processing by outcome type in the devaluation-nondevaluation difference signal. Topographical plots represent time series of *F*-values at each channel at the respective time point. Stars indicate electrodes included in the cluster

Outcome-dependent processing differences between the devaluation and nondevaluation condition were reflected in a significant effect of outcome type on the difference signal (*p* < 0.001), which indicates an interaction between the two factors involved. This effect manifested in two clusters in the P300 latency range: one cluster occupied a time window from 300 to 470 ms at frontal, frontocentral, central, centroparietal, and parietal sites. Frontocentral electrodes were most consistently involved in this cluster. The second, later cluster spanned (fronto)central, central, centroparietal, and parietal electrodes in a time window between 480 and 600 ms. Here, central, centroparietal, and parietal sites were most consistently involved. The two clusters approximate the spatiotemporal distribution of P3a and b (for topographical plots, see Fig. 5 or Supplementary Figure S5 for a more detailed version).

To understand how this interaction effect manifested within valuation conditions, we conducted cluster-based analyses based on *F*-tests with outcome type as the only factor, separately for the devaluation and nondevaluation conditions. In the nondevaluation condition, we found an effect of outcome type (*p* < 0.001), which was evident in one large cluster spanning both time windows implicated in the interaction effect *F*-test (see Supplementary Analysis 2 for the complete results of the separate analysis of the nondevaluation condition). This effect was found at frontal, frontocentral, central, centroparietal, and parietal sites (see Supplementary Figure S6 for topographical plots).

Follow-up *t*-test-based cluster analyses yielded significant differences between all outcome types: the comparison between HPO and LPO showed a significantly more positive amplitude for HPO over LPO (*p* < 0.001). This effect manifested within one large cluster spanning the entire time window of the previously described cluster relating to the general outcome type effect within the nondevaluation condition and largely coincided with the therein implicated electrode sites. The MPO also yielded significantly more positive amplitudes compared to the LPO (*p* < 0.001). This difference was found in two clusters approximating the time windows of the cluster analysis relating to the general outcome type effect within the nondevaluation condition. The later cluster (500-600 ms) included frontal, frontocentral, and central electrode sites, whereas the earlier cluster (320-490 ms) additionally covered centroparietal sites. For the comparison between the HPO and MPO, amplitude showed to be more positive for HPO compared with MPO (*p* = 0.003). Two clusters corresponded to this effect, of which the first one covered the early time window (300-420 ms), and frontal, frontocentral, central, centroparietal, and parietal electrodes. The second cluster coincided with the second cluster (480-590 ms), covering frontocentral, central, centroparietal, and parietal electrodes. Topographical plots can be found in Supplementary Figures S7-9, respectively.

For the devaluation condition, the effect of outcome type emerged as well (*p* < 0.001). This effect showed in one large cluster (300-590 ms) spanning both time windows found in the difference signal cluster analysis over frontal, frontocentral, central, and (centro-)parietal electrode sites (see Supplementary Figure S10 for topographical plots). Follow-up, *t*-test-based cluster analyses revealed more positive amplitudes for the HPO (*p* < 0.001) and MPO (*p* < 0.001) compared with LPO. Importantly, the MPO showed a significantly more positive amplitude compared to the HPO (*p* = 0.017), which is the main difference in the pattern of outcome processing between the devaluation and nondevaluation condition and probably drives the interaction effect. For the comparison between HPO and LPO, a large cluster covering the entire early time window and early parts of the late time window was found (300-540 ms), including frontal, frontocentral, central, and centroparietal electrodes. A similar cluster was found for the comparison between MPO and LPO, covering again both time windows (300-600 ms) and including frontal, frontocentral, central, and centroparietal electrode sites. Concerning the comparison between HPO and MPO, a comparatively small cluster emerged (340-400 ms) covering frontal, frontocentral, central, centroparietal, and parietal electrodes. Topographical plots can be found in Supplementary Figures S11-13, respectively.

Taken together, a general outcome type effect over both valuation conditions was found in the time window of the P2, FRN, and P300, where the amplitude for the two rewarding outcomes (HPO and MPO) differed from the amplitude for LPO but did not differentiate between HPO and MPO. Within the nondevaluation condition, ERP amplitudes in the time window of the P300 reflected the preference structure of outcome types (HPO > MPO > LPO). In contrast, differentiation between HPO and MPO was reversed in the devaluation condition, with higher amplitudes for MPO compared with HPO, whereas differentiation of these two more preferred outcome types and LPO was preserved.

## Discussion

This study investigated whether the electrophysiological representation of subjective reward preferences is manipulable through selective devaluation in the context of a gambling task. Participants completed a task in which they could win one of three outcomes for which they had an individual high, medium, or low preference. Each participant was tested in two separate sessions one of which entailed selective devaluation of the preferred outcome (HPO) by means of ad libitum consumption. The other test session was completed on an empty stomach and served as control condition. Desire-to-eat ratings confirmed the presence of individual outcome preferences as well as the selective devaluation effect for the HPO. We found that subjective preferences were coded in the ERP, with more positive amplitudes for more preferred outcomes. Preference differences between LPO and HPO as well as LPO and MPO but not between MPO and HPO were reflected in the time windows of N1, P2, late FRN, and P300, when valuation condition was not considered. Differences in outcome processing between valuation conditions reflected the spatiotemporal shift of P3a and b. While in the nondevaluation condition, preference succession as reflected in desire-to-eat ratings was coded in the ERP amplitude (HPO > MPO > LPO), selective devaluation reduced the amplitude in the P300 time window for the HPO, so that it was significantly less positive than the MPO (although still significantly more positive than the LPO).

In accordance with studies showing the P300’s sensitivity to outcome magnitude (Bellebaum, Polezzi et al., 2010; Gu et al., 2011; Kreussel et al., 2012; Meadows et al., 2016; Wu & Zhou, 2009), amplitude in the P300 time window was modulated by outcome type. Of note, while we did not find a differentiation between HPO and MPO when ERPs were averaged across valuation conditions, it is conceivable that this differentiation was obscured by the interaction effect, which also coincided with the P300 time window. Indeed, in the nondevaluation condition, amplitudes in the P300 time window distinguished between all three outcome types. The present results together with earlier findings (Peterburs et al., 2019) thus corroborate the notion that the P300 codes the motivational value of an outcome (Begleiter et al., 1983; Nieuwenhuis et al., 2005; San Martín, 2012; Severo et al., 2020). Future studies will need to investigate whether this motivational value remains reward type-specific or represents a common value with the ability to compare a multitude of reward types (Bartra et al., 2013). Additionally, it is still unclear how the subjective value is computed. A study by Suzuki et al. (2017) showed that activity in lateral OFC associated with subjective values for food items scaled with subjective estimates about their nutritional attributes, especially attributes such as fat, carbohydrate, protein, and vitamin content. Investigating whether this finding extends to preference effects in the ERP could help to elucidate the spatiotemporal dynamics of how feedback evaluation and possibly also decision-making processes come about, e.g., which reward attributes are coded in early ERP components and which ones are evaluated only at later processing stages.

In line with previous work showing that the P2 is sensitive to reward magnitude (Flores et al., 2015; San Martín et al., 2010) and distinguishes whether a reward was given (Potts et al., 2006), amplitude in the P2 time window was modulated by outcome type as well. While the lack of differentiation between HPO and MPO cannot be explained by masking through the interaction effect, Peterburs et al. (2019), who did not perform a manipulation of motivational state, also found precisely this pattern. They suggested that preference coding in the P2 seems to be a first, quick outcome evaluation based on mere salience to guide attention (Potts, 2004). The present findings are well in line with this. The effect in this cluster started as early as 90 ms after outcome onset, thus including the time window of the N1, which is a component also linked to attentional processes (Boksem et al., 2005; Gonzalez et al., 1994; Hillyard & Anllo-Vento, 1998) that has been implicated in feedback processing as well (Mathias et al., 2017).

Concerning the FRN time window, we found preference effects only for late portions of the FRN. The observed patterns are largely consistent with studies that found outcome magnitude effects in the FRN (Gu et al., 2011; Holroyd et al., 2004; Kreussel et al., 2012; Meadows et al., 2016; Wu & Zhou, 2009), except for the lack of differentiation between HPO and MPO, which was unexpected given the findings by Peterburs et al. (2019), who used a highly similar experimental design and found differentiation between HPO and MPO in the FRN time window. This cannot be attributed to masking by the interaction effect, as evident when considering the full clusters in the outcome type analysis for the nondevaluation condition (see Supplementary Analysis 2). A possible explanation for this unexpected finding might be the smaller sample size in the present study compared with the previous one by Peterburs et al. Additionally, the difference between HPO and MPO preference ratings was larger in the study by Peterburs et al. compared with the current study. It seems possible that the HPO-MPO desire-to-eat difference might not have been large enough to show effects in the FRN time window in the current study. Lastly, while our experimental design was highly similar to that used in the earlier study, Peterburs et al. used only three different snack items in total and fitted the HPO and MPO from only two, possibly rendering the preference structure aspect more obvious to participants. In the current study, the availability of nine snack items might have obscured the logic behind the choices of stimuli shown in the experimental task. These methodological differences might help to explain the divergent result patterns in terms of effects of subjective outcome probability expectations that might have emerged based on participants’ knowledge about the preference structure but not outcome randomness in the previous but not the current study. Indeed, subjective expectations can differ substantially from objective outcome probabilities (Hajcak et al., 2005; Hajcak et al., 2007). However, as neither the previous nor the present study assessed subjective expectations, this explanation remains rather speculative. Future studies should investigate the effect of subjective expectations in the context of preference coding in the outcome-locked ERP.

Interestingly, we failed to find modulations in the FRN time window by outcome type as a function of valuation condition and thus cannot confirm the findings by Baker et al. (2016). Instead, we found a selective devaluation effect of the HPO in the latency range of the P300, which is consistent with both the notion that the P300 represents the motivational value of stimuli (Begleiter et al., 1983; Nieuwenhuis et al., 2005; San Martín, 2012; Severo et al., 2020) and the notion that the P300 codes attentional processes (Polich, 1987). While in the nondevaluation condition, the HPO has the highest motivational value for consumption after the experimental task, its value substantially decreases after consumption to satiety, as also reflected in the desire-to-eat ratings. However, it can similarly be argued that the HPO has the highest salience in the nondevaluation condition and salience is reduced in the devaluation condition, as valuation and salience are perfectly correlated in a context where only reward but no punishment is applied (Kahnt, 2018). While we did not consider valence but regarded the outcomes as distinguishable simply by their difference in (subjective) magnitude/preference, it could be argued that the selectively devalued HPO becomes a rather negative item, which is avoided after devaluation, as studies with behavioural choice measures have found (Howard & Kahnt, 2017; Valentin et al., 2007). Thus, we suggest that the present findings in the P300 time window speak rather for a coding of motivational value than salience, but future studies will need to capture the distinction of valence and magnitude more closely, possibly by including the factor valence into the analysis and using punishments in addition to rewards.

The observed selective devaluation effect for the HPO is also consistent with fMRI studies reporting selective devaluation effects in the OFC (Gottfried et al., 2003; Howard & Kahnt, 2017; Kringelbach et al., 2003; O'Doherty et al., 2000; Valentin et al., 2007), which speaks for a common coding of valuation and desirability in P300 and OFC. This is also in agreement with studies connecting P300 with the OFC via LORETA source localisation (Andreou et al., 2013; Rule et al., 2002) and lesion studies (Rule et al., 2002). Our study further demonstrates that selective devaluation works in a context with more than two outcomes and leaves the processing of both non-devalued outcomes unchanged. An additional explorative analysis investigating the selective devaluation effect in the P300 additionally hinted toward a reduced devaluation with rising BMI (see Supplementary Analysis 1), which would be in agreement with studies showing a reduced behavioural selective devaluation effect with rising BMI (Horstmann et al., 2015; Janssen et al., 2017), although future studies need to confirm this effect due to the unobjective quantification of BMI in our study and the explorative nature of this analysis (de Groot, 2014).

## Conclusions and Outlook

We provide first evidence that effects of selective devaluation are reflected in an ERP component associated with outcome processing, i.e., the P300. By using a within-subject design with stimuli carefully matched in desire-to-eat ratings and with the same stimuli throughout valuation conditions for each participant, these findings can likely be explained in terms of changes in the motivational significance for the respective outcomes. The present results suggest that the P300 might be an especially interesting measure to investigate the mechanisms leading to overeating and obesity. Nijs et al. (2010) reported that overall satiety manifests differently for overweight and obese compared to lean subjects: while for lean subjects, the P300 decreased with satiety, the opposite pattern was found for overweight and obese subjects. We speculate that this effect can be replicated for the selective devaluation paradigm with a sufficient sample size of overweight participants, consistent with behavioural findings of reduced goal-directed behaviour with increasing BMI (Horstmann et al., 2015; Janssen et al., 2017). Especially interesting would be whether this maladaptive behaviour is reversible. Soares et al. (2012) showed that stress-induced shifts towards habitual instead of goal-directed behaviour and associated network activations vanished after a stress-free period of six weeks. To examine whether this reversibility extends to obesity-related processes, longitudinal studies which control for participants’ diet and BMI in relation to neurophysiological measures associated with valuation, such as the P300 or the activation of the OFC, are needed, especially in the light of rising obesity rates worldwide (Chooi et al., 2019).

## Supplementary Information

ESM 1 (PDF 4307 kb)

## Data Availability

The data that support these findings are openly available through the Open Science Framework at https://doi.org/10.17605/OSF.IO/UWZET.

## Declarations

### Code Availability

All templates and scripts used to preprocess and analyse data are available through the Open Science Framework together with the data they are applied on (see above).
