# Supplementary material for: Selective Devaluation Affects the Processing of Preferred Rewards
Source: Cogn Affect Behav Neurosci. 2021 Apr 30;21(5):1010–25. doi: 10.3758/s13415-021-00904-x (PMC8455391; doi:10.3758/s13415-021-00904-x)
Supplement: Supplementary file 1 — (PDF 4307 kb) [file 13415_2021_904_MOESM1_ESM.pdf]

Selective Devaluation Affects the Processing of Preferred Rewards

Dana M. Huvermann[1], Christian Bellebaum[1], Jutta Peterburs[1, 2]

[1]Department of Biological Psychology, Heinrich-Heine-University Düsseldorf, Germany

[2]Department of Medicine, Medical Psychology, MSH Medical School Hamburg

Supporting Information

Supplementary Analysis 1: Link between BMI and selective devaluation effects 3

Supplementary Figure S1: Correlation between BMI and cluster amplitude 4

Supplementary Analysis 2: Replication of outcome type effect reported in Peterburs et al. (2019) 5

Supplementary Figure S2: Outcome type main effect 7

Supplementary Figure S3: Effect of HPO over LPO for average amplitudes 8

Supplementary Figure S4: Effect of MPO over LPO for average amplitudes 9

Supplementary Figure S5: Interaction effect of outcome type and valuation condition 10

Supplementary Figure S6: Outcome type effect in the nondevaluation condition 11

Supplementary Figure S7: Effect of HPO over LPO in the nondevaluation condition 12

Supplementary Figure S8: Effect of MPO over LPO in the nondevaluation condition 13

Supplementary Figure S9: Effect of HPO over MPO in the nondevaluation condition 14

Supplementary Figure S10: Outcome type effect in the devaluation condition 15

Supplementary Figure S11: Effect of HPO over LPO in the devaluation condition 16

Supplementary Figure S12: Effect of MPO over LPO in the devaluation condition 17

Supplementary Figure S13: Effect of MPO over HPO in the devaluation condition 18

References 19

Supplementary Analysis 1

In light of studies by Horstmann et al. (2015) and Janssen et al. (2017) who found a decreased behavioural selective devaluation effect with higher BMI, we conducted an exploratory correlational analysis between the BMI and the selective devaluation effect. For the selective devaluation effect, we used the mean amplitude of the devaluation-nondevaluation difference signal for the HPO outcome in the time windows yielded by the cluster-based analysis for the difference signal (300-470 and 480-600 ms) for electrodes Fz, FCz, Cz, and Pz. This analysis yielded two significant correlations, one at electrode site Cz for the early cluster time window (r = .37, p = .042) and the second at FCz for the late cluster time (r = .36, p = .045). Complete inferential statistics and descriptive data can be found in Figure S1 below. These correlations are consistent with a decreased selective devaluation effect in the P300 with increasing BMI. As we retrieved the BMI from participants' statements of height and weight, which are known to be imprecise (Spencer, Appleby, Davey, & Key, 2002), and since the present sample included only few overweight (n = 7) and obese participants (n = 2), future studies will need to explore whether this effect prevails with more objective BMI measures and a better fitting sample.

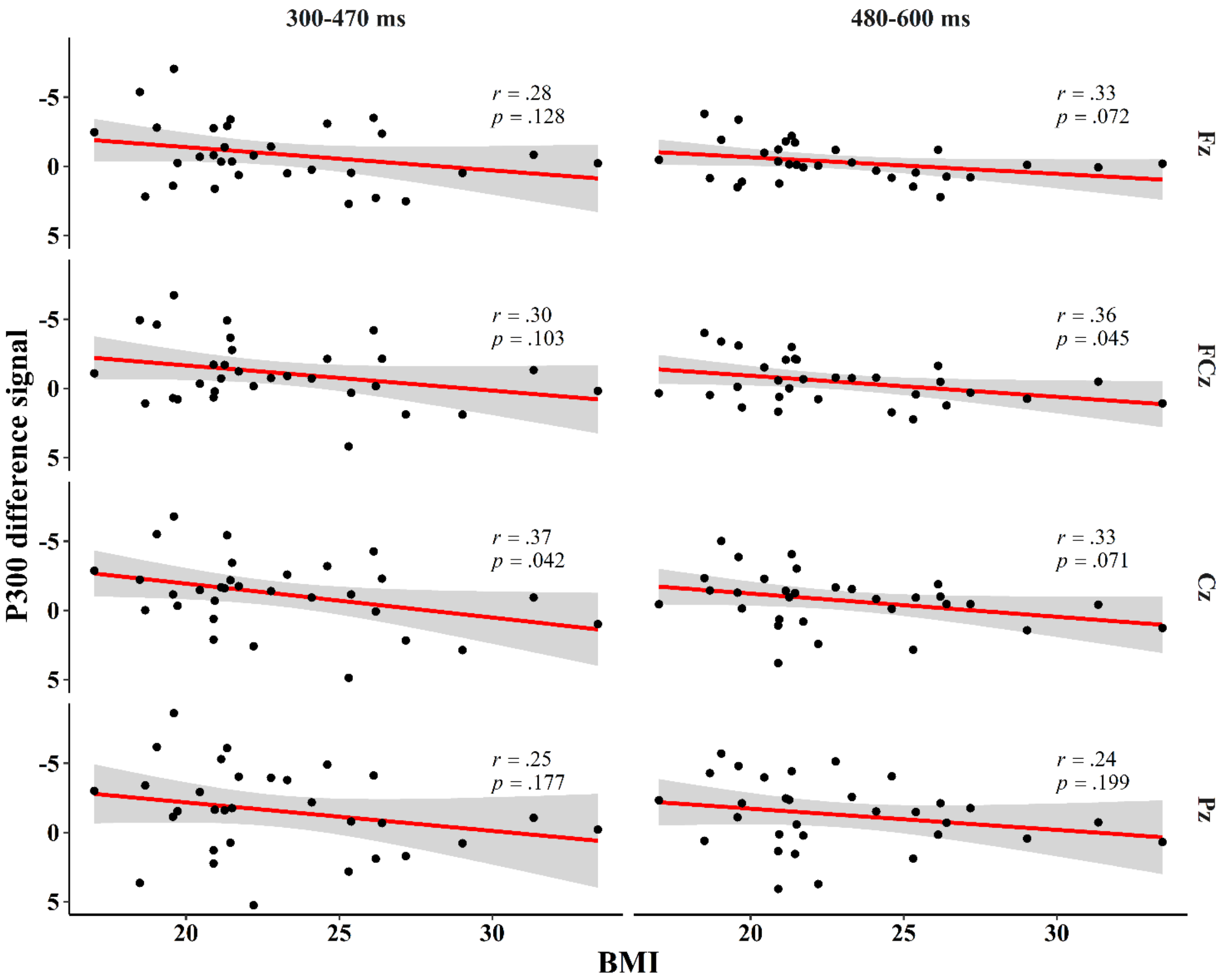

Figure S1. BMI according to subjects' estimation of their weight and height plotted against difference in mean amplitude (devaluation - nondevaluation) in the cluster time windows (300-470 and 480-600 ms) for Fz, FCz, Cz, and Pz. Regression line is plotted in red with a grey ribbon for the confidence interval.

Supplementary Analysis 2

To see whether we could replicate the findings of Peterburs, Sannemann, and Bellebaum (2019), we took into consideration all clusters for the analysis of the outcome type effect in the nondevaluation condition which are reported below.

Cluster-based permutation tests for the outcome type main effect in the nondevaluation condition revealed significant differences within outcome types ($p < .001$; for topographical plots, see Figure S4). This effect was evident in two clusters, of which the earlier cluster covered a time window from 150 to 200 ms and included frontal, frontocentral, central, centroparietal, and parietal electrodes. This cluster roughly corresponds to spatio-temporal attributes of the P2. The later cluster covered a time window ranging from 250 to 600 ms and spanned over frontal, frontocentral, central, centroparietal, and parietal electrodes. Frontocentral and central electrodes were most consistently included, suggesting that this cluster captures late portions of the FRN and the P300.

Follow-up cluster-based analyses with dependent-sample *t*-tests yielded significant differences between all three outcome types. The HPO ($p < .001$) and MPO ($p < .001$) yielded significantly more positive amplitudes compared to the LPO. Differences between the HPO and LPO manifested in two clusters within the time windows from 150 to 200 ms, and from 250 to 600 ms, spanning over frontal, frontocentral, central, centroparietal, and parietal electrodes (see Figure S5). For the MPO compared to the LPO, an earlier cluster covered the time window between 150 and 190 ms and included frontal, frontocentral, central, centroparietal, and parietal electrodes. Two later clusters emerged of which one covered the time window from 320 to 490 ms and the other the time window from 500 to 600 ms. Both clusters included frontal, frontocentral, and central electrodes and the first time window additionally included centroparietal electrodes inconsistently (see Figure S6).

Amplitudes were also significantly more positive for the HPO compared to the MPO

(p = .003). This effect was evident in clusters covering only later time windows. One cluster covered a time window between 300 and 420 ms at frontal, frontocentral, central, centroparietal, and parietal electrodes, while a second cluster was found from 480 and 590 ms at frontocentral, central, centroparietal, and parietal sites (see Figure S7).

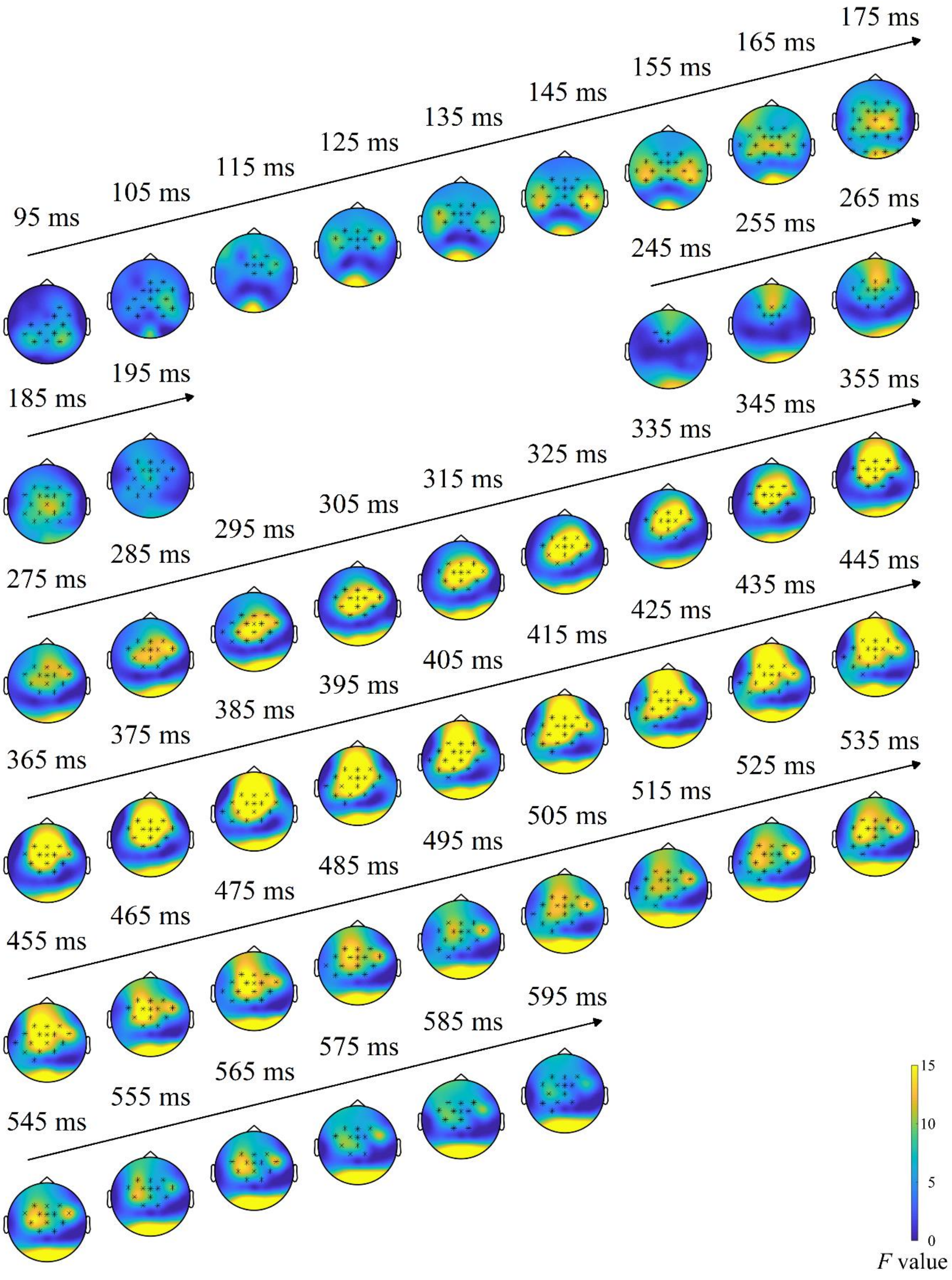

Figure S2. Clusters (90-200 and 240-600 ms) showing significant modulation of outcome processing by outcome type. Topographical plots represent time series of *F*-values at each time point and channel. Stars indicate electrodes included in the cluster.

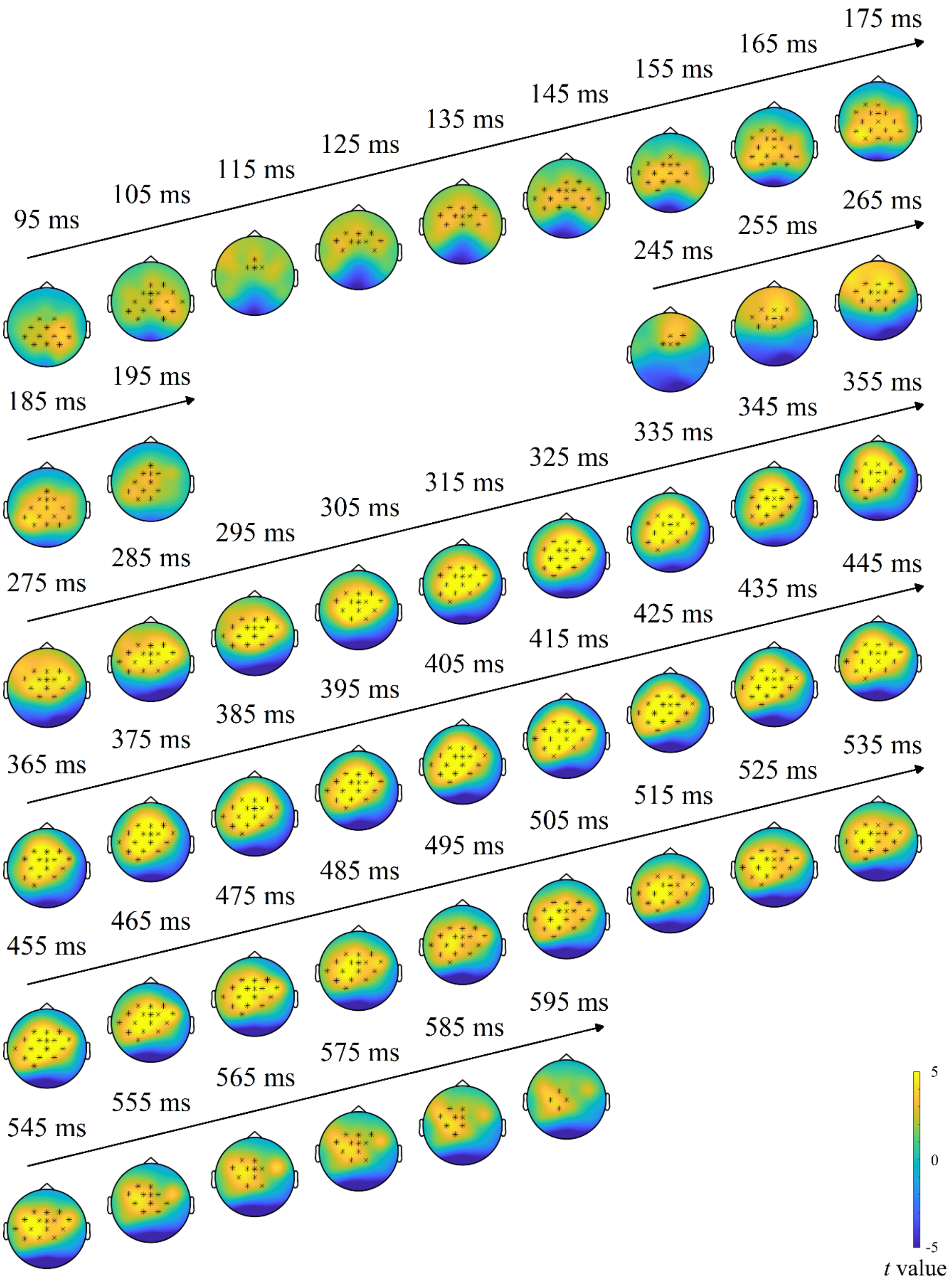

Figure S3. Clusters (90-200 and 240-600 ms) showing more positive amplitudes for the high preference outcome (HPO) over the low preference outcome (LPO) for the mean amplitude between the devaluation and nondevaluation condition. Topographical plots represent time series of *t*-values at each time point and channel. Stars indicate electrodes included in the cluster.

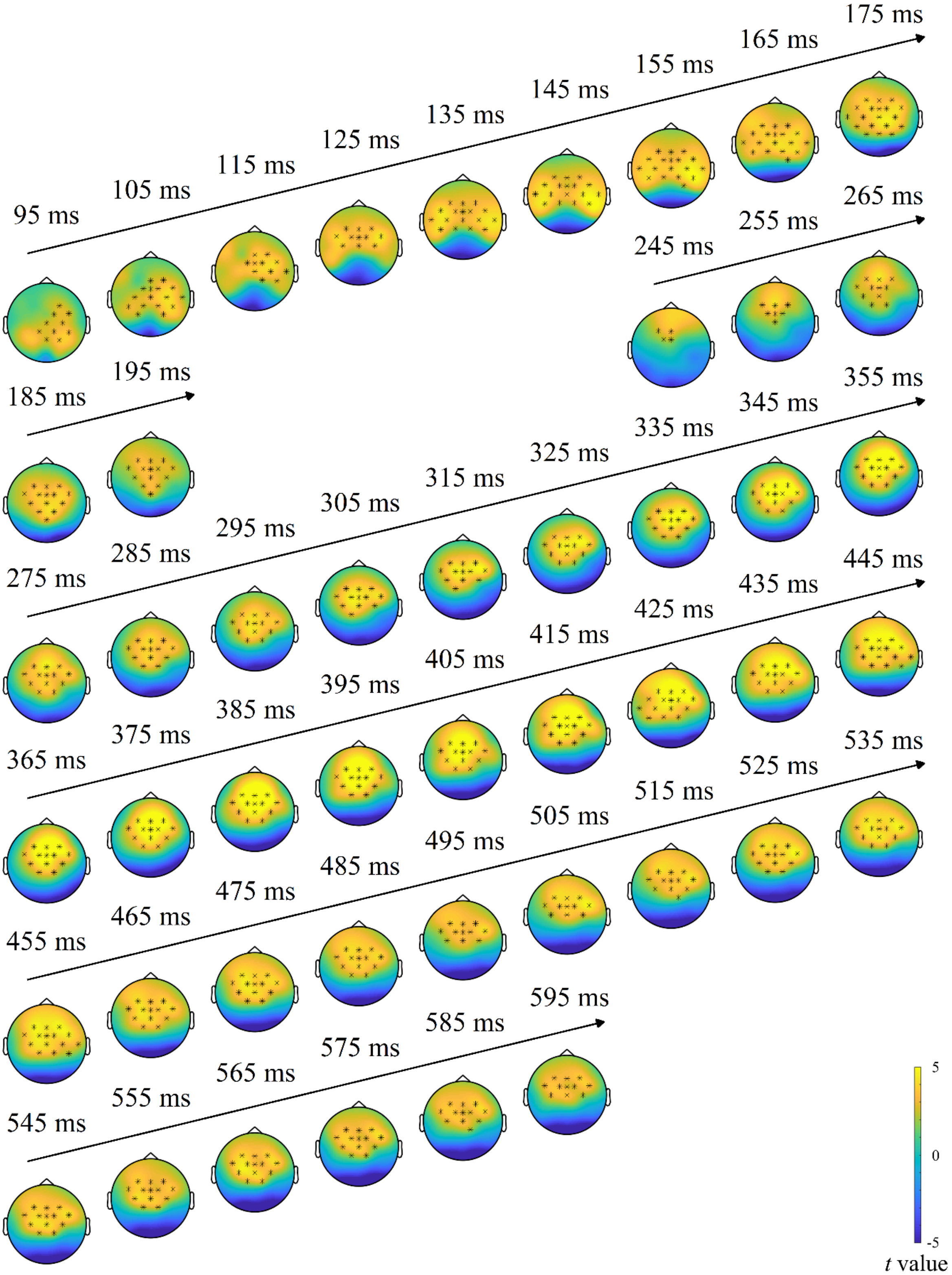

Figure S4. Clusters (90-200 and 240-600 ms) showing more positive amplitudes for the medium preference outcome (MPO) over the low preference outcome (LPO) for the mean amplitude between the devaluation and nondevaluation condition. Topographical plots represent time series of *t*-values at each time point and channel. Stars indicate electrodes included in the cluster.

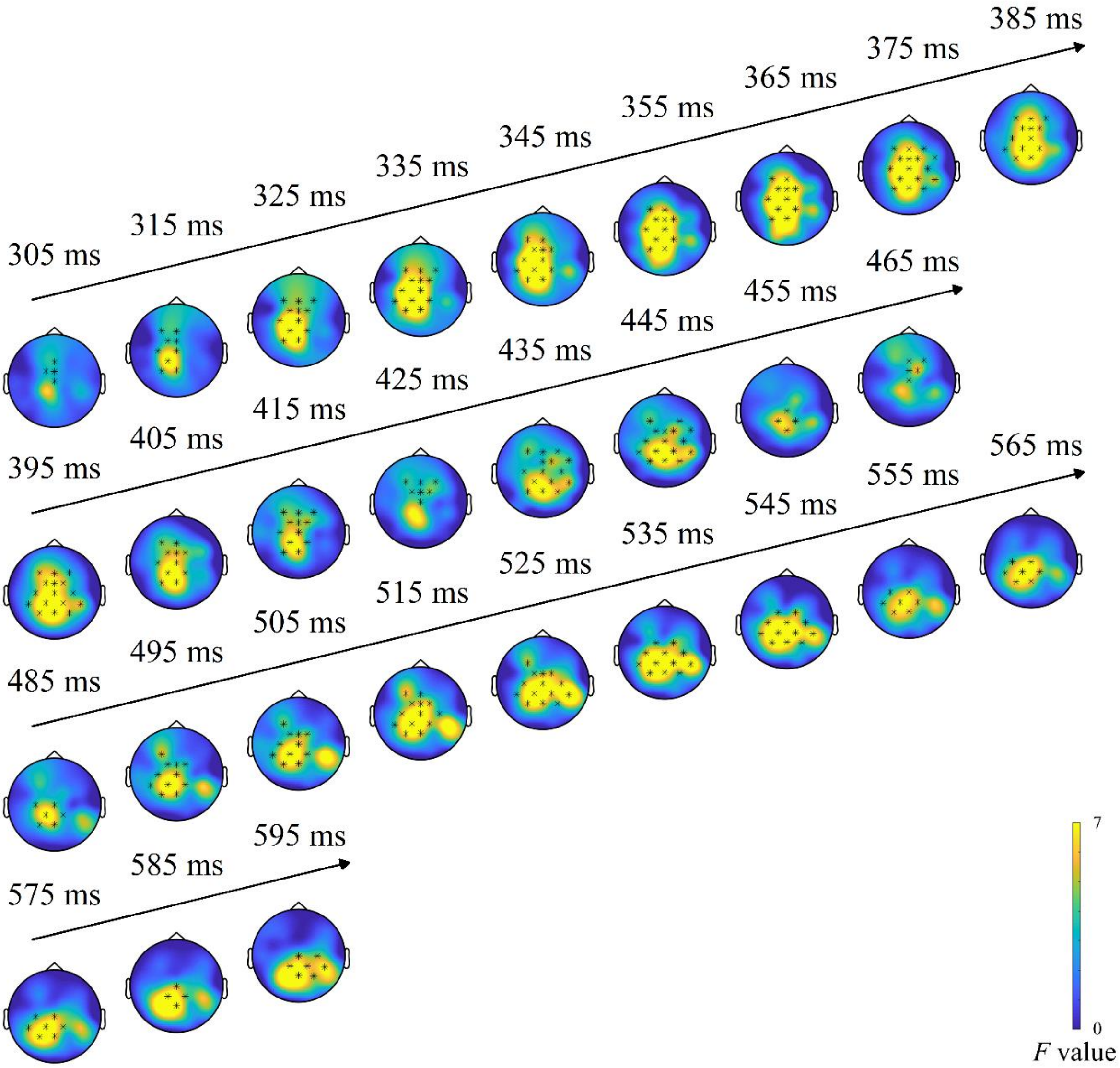

Figure S5. Clusters (300-470 and 480-600 ms) showing significant modulation of outcome processing by outcome type in the devaluation-nondevaluation difference signal. Topographical plots represent time series of *F*-values at each time point and channel. Stars indicate electrodes included in the cluster.

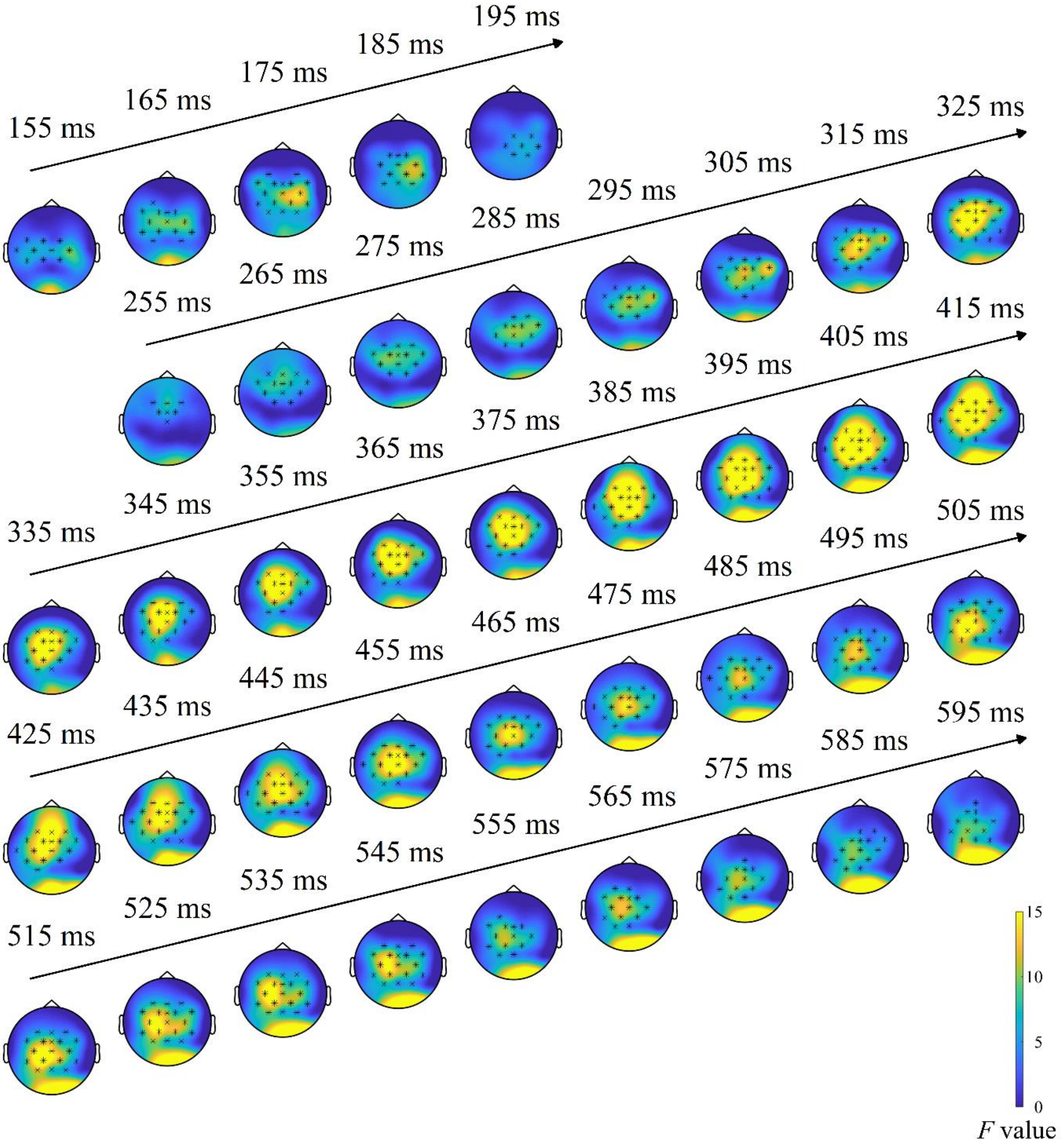

Figure S6. Clusters (150-200 and 250-600 ms) showing an effect of outcome type in the nondevaluation condition. Topographical plots represent time series of *F*-values at each time point and channel. Stars indicate electrodes included in the cluster.

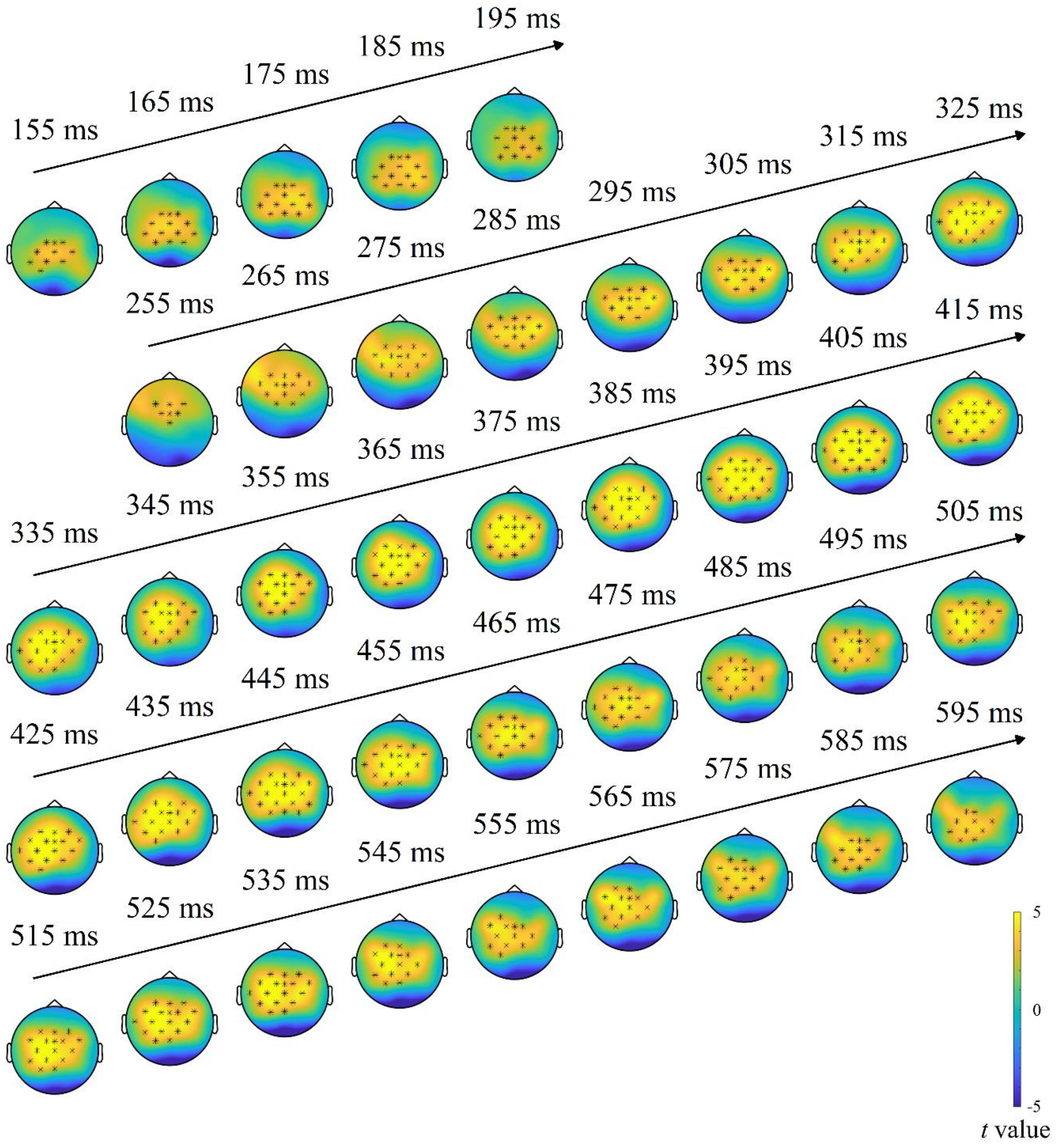

Figure S7. Clusters (150-200 and 250-600 ms) showing more positive amplitudes for the high preference outcome (HPO) over the low preference outcome (LPO) for the nondevaluation condition. Topographical plots represent time series of *t*-values at each time point and channel. Stars indicate electrodes included in the cluster.

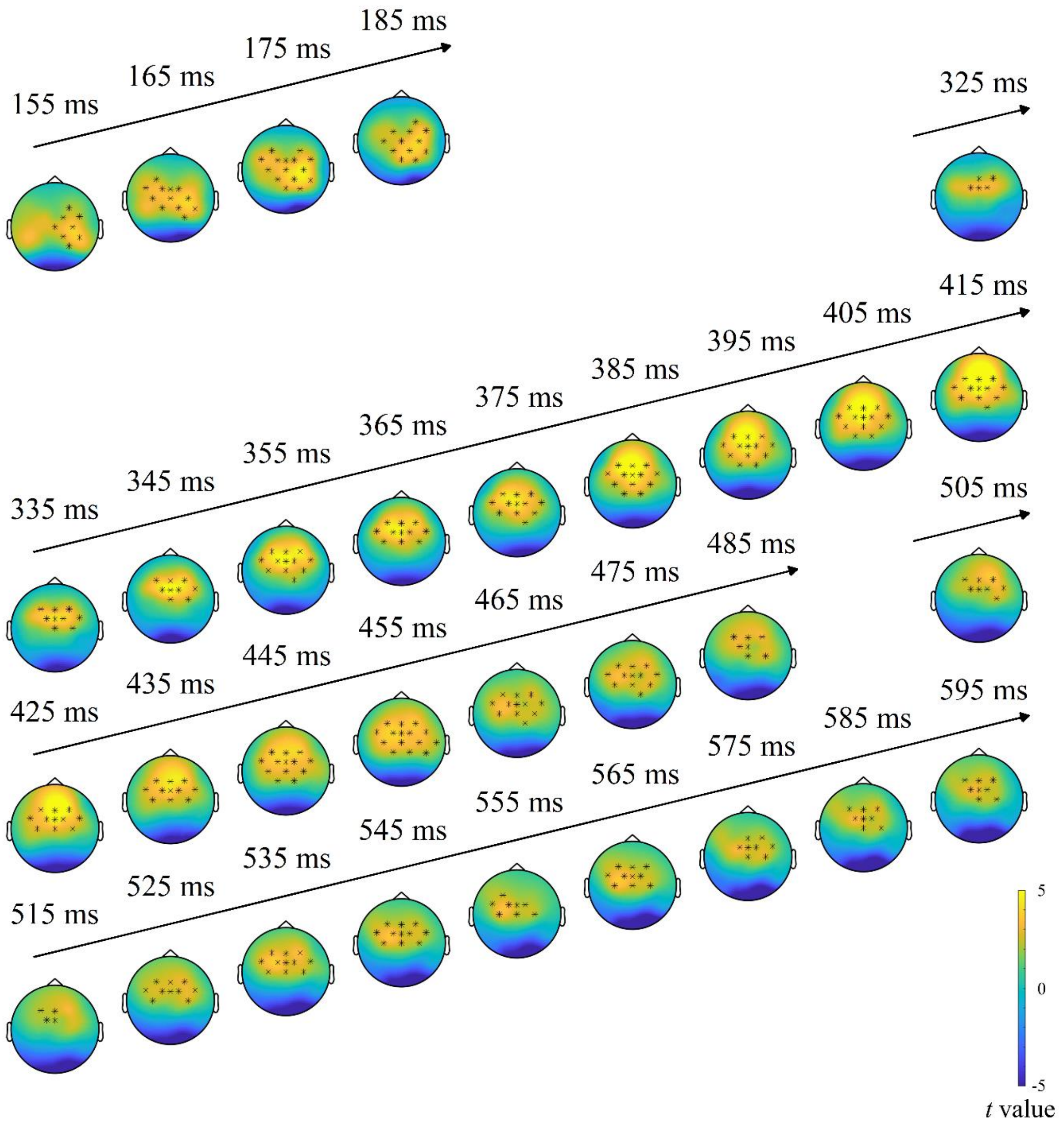

Figure S8. Clusters (150-190, 320-490, and 500-600 ms) showing more positive amplitudes for the medium preference outcome (MPO) over the low preference outcome (LPO) for the nondevaluation condition. Topographical plots represent time series of *t*-values at each time point and channel. Stars indicate electrodes included in the cluster.

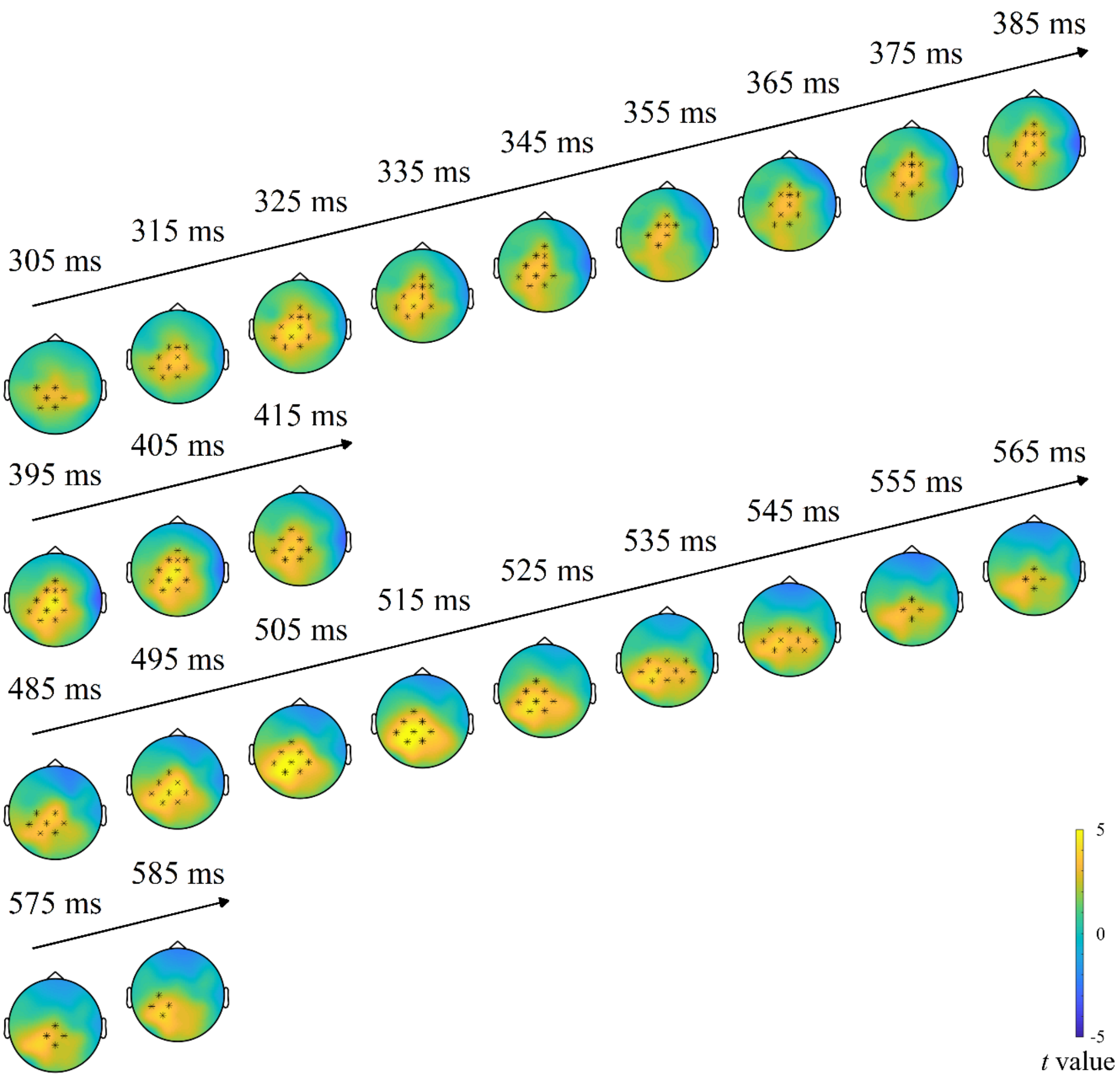

Figure S9. Clusters (300-420 and 480-590 ms) showing more positive amplitudes for the high preference outcome (HPO) over the medium preference outcome (MPO) for the nondevaluation condition. Topographical plots represent time series of *t*-values at each time point and channel. Stars indicate electrodes included in the cluster.

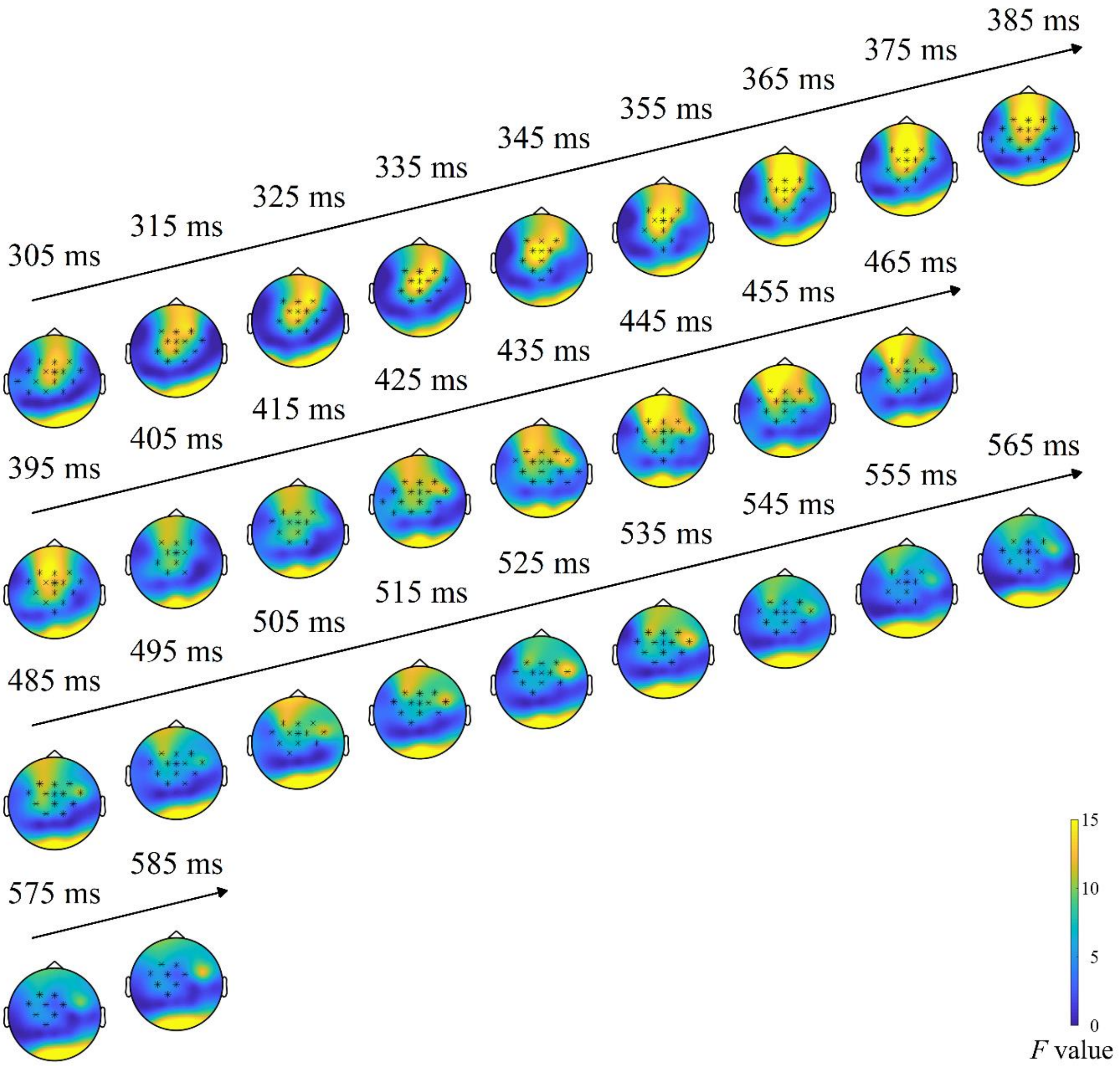

Figure S10. Clusters (300-470 and 480-590 ms) showing an effect of outcome type in the devaluation condition. Topographical plots represent time series of *F*-values at each time point and channel. Stars indicate electrodes included in the cluster.

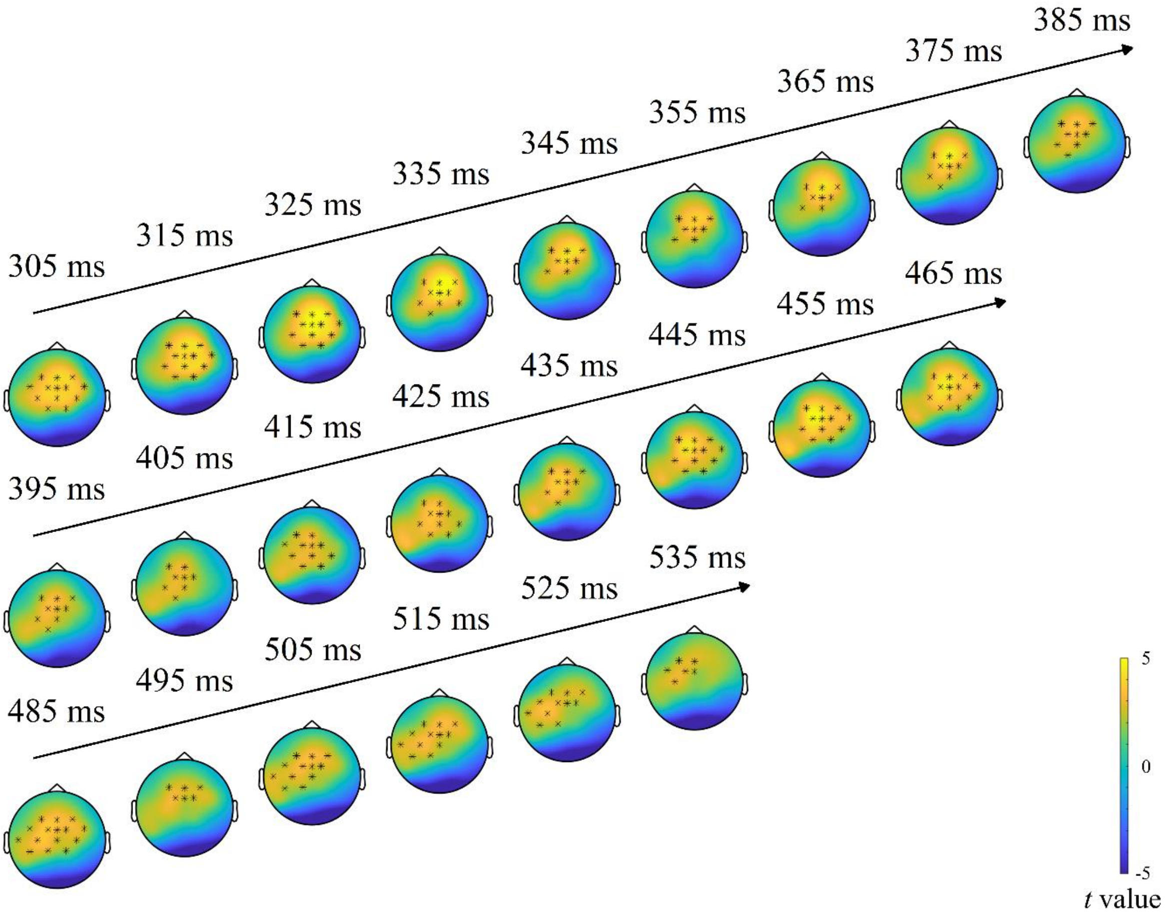

Figure S11. Clusters (300-470 and 480-540 ms) showing more positive amplitudes for the high preference outcome (HPO) over the low preference outcome (LPO) for the devaluation condition. Topographical plots represent time series of *t*-values at each time point and channel. Stars indicate electrodes included in the cluster.

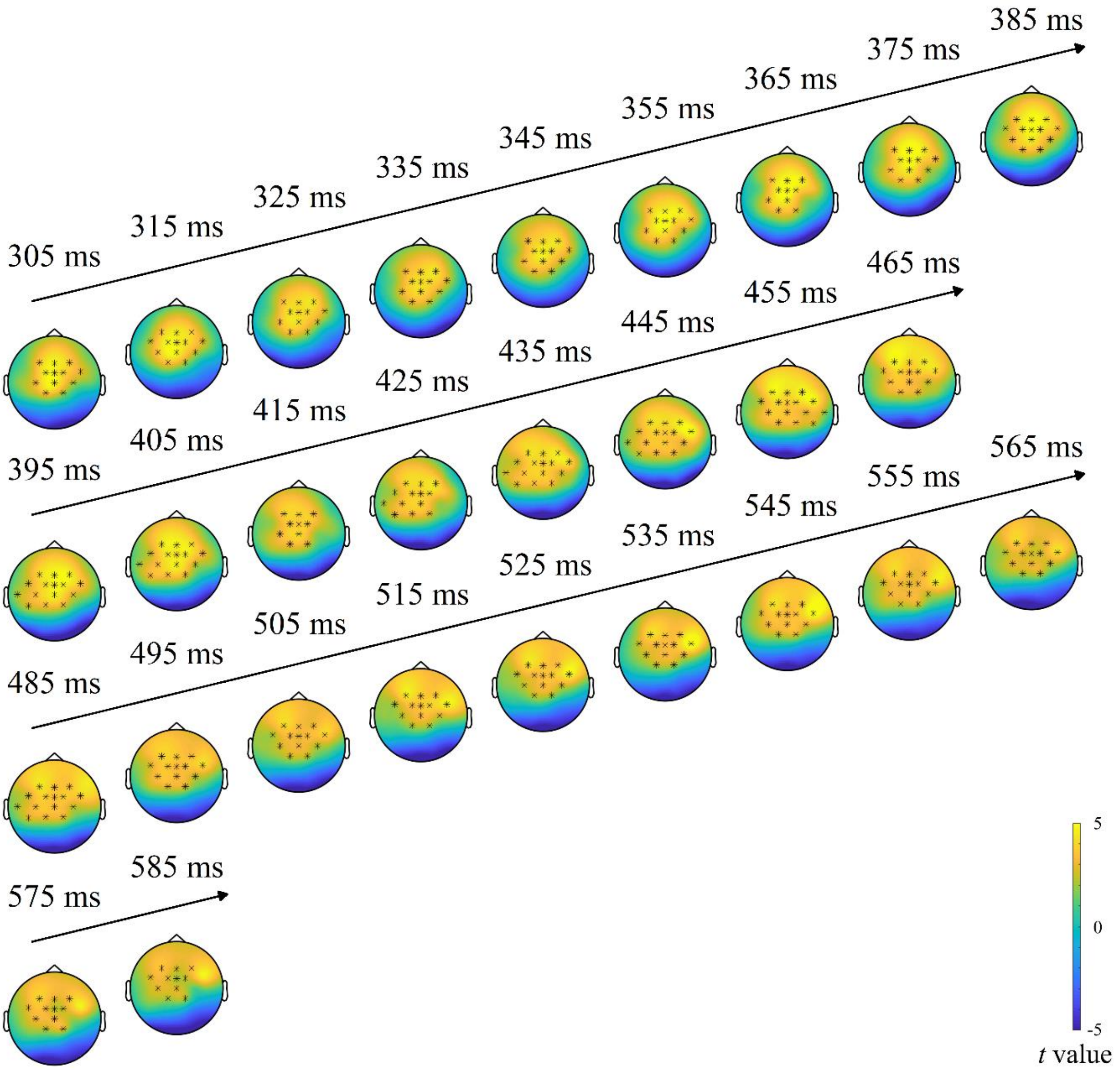

Figure S12. Clusters (300-470 and 480-590 ms) showing more positive amplitudes for the medium preference outcome (MPO) over the low preference outcome (LPO) for the devaluation condition. Topographical plots represent time series of *t*-values at each time point and channel. Stars indicate electrodes included in the cluster.

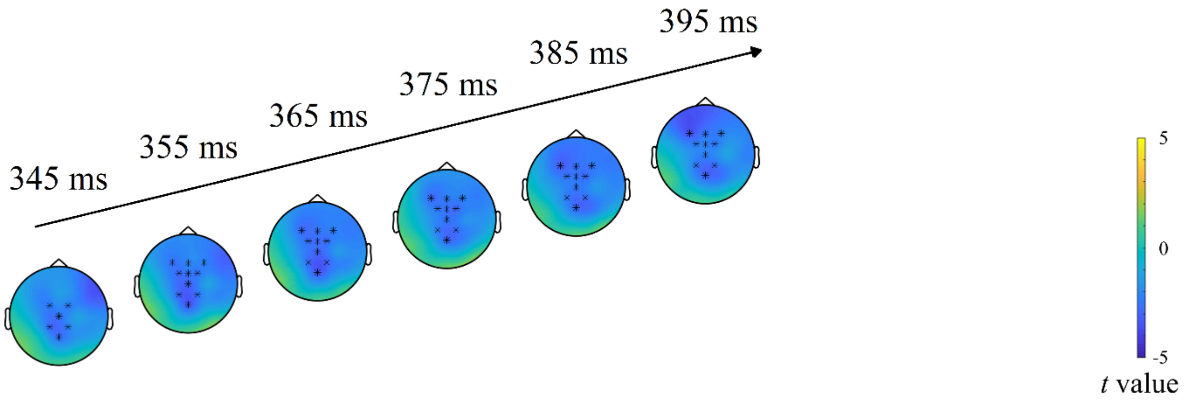

Figure S13. Cluster (340-400 ms) showing more positive amplitudes for the medium preference outcome (MPO) over the high preference outcome (HPO) for the devaluation condition. Topographical plots represent time series of *t*-values at each time point and channel. Stars indicate electrodes included in the cluster.

References

Horstmann, A., Dietrich, A., Mathar, D., Pössel, M., Villringer, A., & Neumann, J. (2015). Slave to habit? Obesity is associated with decreased behavioural sensitivity to reward devaluation. *Appetite, 87*, 175-183. doi:10.1016/j.appet.2014.12.212

Janssen, L. K., Duif, I., van Loon, I., Wegman, J., de Vries, J. H., Cools, R., & Aarts, E. (2017). Loss of lateral prefrontal cortex control in food-directed attention and goal-directed food choice in obesity. *NeuroImage, 146*, 148-156. doi:10.1016/j.neuroimage.2016.11.015

Peterburs, J., Sannemann, L., & Bellebaum, C. (2019). Subjective preferences differentially modulate the processing of rewards gained by own vs. observed choices. *Neuropsychologia, 132*, 107139. doi:10.1016/j.neuropsychologia.2019.107139

Spencer, E. A., Appleby, P. N., Davey, G. K., & Key, T. J. (2002). Validity of self-reported height and weight in 4808 EPIC–Oxford participants. *Public Health Nutrition, 5*(4), 561-565. doi:10.1079/PHN2001322
